# N6-methyladenosine–mediated up-regulation of ARRB2 regulates intrahepatic cholangiocarcinoma malignant progression and pemigatinib resistance through MAPK and Hippo signaling pathways

**DOI:** 10.1038/s41419-026-08574-8

**Published:** 2026-04-15

**Authors:** Haoqi Chen, Xiaowen Wang, Wenfeng Zhu, Wenjie Zheng, Qiwei Yang, Zhixing Liang, Yuan Zhang, Xuejiao Li, Jinliang Liang, Xiaolong Chen, Hua Li, Linsen Ye, Hui Li, Xijing Yan, Shuguang Zhu, Genshu Wang

**Affiliations:** 1 Department of Hepatic Surgery, Liver Transplantation, The Third Affiliated Hospital of Sun Yat-Sen University, Guangzhou, China; 2 Guangdong Key Laboratory of Liver Disease Research, The Third Affiliated Hospital of Sun Yat-Sen University, Guangzhou, China; 3 Department of Breast and Thyroid Surgery, Lingnan Hospital, The Third Affiliated Hospital of Sun Yat-Sen University, Guangzhou, China; 4 Department of Hepatobiliary and Pancreatic Surgery, The Second Affiliated Hospital of Guangzhou Medical University, Guangzhou, China; 5 Department of Vascular Surgery, Zhejiang Provincial People’s Hospital (Affiliated People’s Hospital), Hangzhou Medical College, Hangzhou, China; 6 Department of Hepatic Surgery and Liver Transplantation, Guangdong Provincial Hospital of Traditional Chinese Medicine, Guangzhou, China; 7 Department of Pathology, Guangdong Provincial Hospital of Traditional Chinese Medicine, Guangzhou, China; 8 Department of Hepatobiliary Surgery, The First Affiliated Hospital of Jinan University, Guangzhou, China; 9 Department of Hepatobiliary Pancreatic Tumor Center, Chongqing University Cancer Hospital, Chongqing, China; 10 Chongqing Key Laboratory for the Mechanism and Intervention of Cancer Metastasis, Chongqing University Cancer Hospital, Chongqing, China; 11 State Key Laboratory of Traditional Chinese Medicine Syndrome, The Second Affiliated Hospital of Guangzhou University of Chinese Medicine, Guangzhou, China

**Keywords:** Cancer, Cancer

## Abstract

Intrahepatic cholangiocarcinoma (ICC) is a major contributor to cancer-related mortality on a global scale, yet it suffers from a lack of reliable early diagnostic biomarkers and effective therapeutic targets. Pemigatinib has been identified as a therapeutic option for advanced ICC; however, its long-term clinical efficacy is significantly hindered by the development of drug resistance. To address this, pemigatinib-resistant ICC cells were established by culturing with increasing drug treatment. 98 pairs of ICC tissue samples were collected and analyzed to assess the association between β-arrestin 2 (ARRB2) and ICC progression. The role and mechanism of ARRB2 in the malignant progression of ICC and resistance to pemigatinib were explored in vitro and in vivo experiments. The results demonstrated that ARRB2 expression is markedly upregulated in pemigatinib-resistant ICC cells compared to their parental counterparts. Suppression of ARRB2 expression markedly attenuated ICC chemoresistance to pemigatinib. Clinical data further verified that ARRB2 is correlated with poorer pathological stage and prognosis in ICC patients. Mechanistic studies revealed that ARRB2 activation in ICC is mediated by METTL3-dependent m6A methylation. Functional analyses demonstrated that ARRB2 promotes the malignant progression of ICC by facilitating YAP nuclear translocation while also modulating the sensitivity of ICC to pemigatinib through the Raf-MEK-ERK signaling axis. This study identifies the tumor-promoting activities of ARRB2 and elucidates the regulatory mechanism of the METTL3-ARRB2-YAP/Raf axis in ICC, which may provide a novel prognostic biomarker and potential therapeutic target for human ICC.

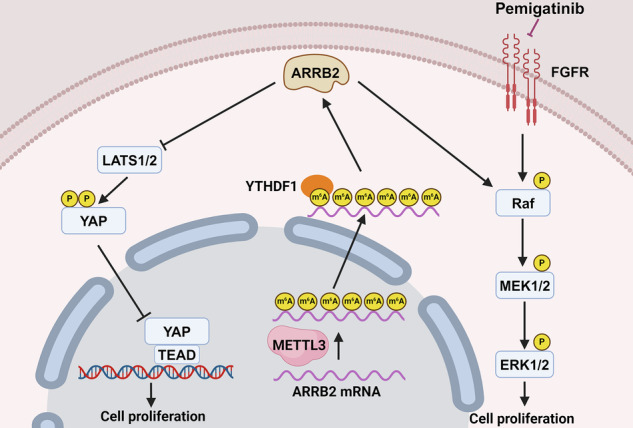

## Introduction

Cholangiocarcinoma (CCA) is a malignant tumor originating from the epithelial cells lining the bile ducts. Anatomically, CCA can be further categorized into intrahepatic (iCCA), perihepatic (pCCA), and distal (dCCA) [1]. These classifications exhibit distinct clinical characteristics, surgical options, prognostic outcomes, and biological behaviors [2]. Intrahepatic cholangiocarcinoma (ICC) is recognized as a primary liver malignancy, second only to hepatocellular carcinoma in prevalence [3]. Surgical resection remains the preferred treatment for ICC; however, due to difficulties in early diagnosis, only 20% to 40% of patients are eligible for surgical resection [4]. In clinical practice, targeted therapies such as Pemigatinib and Futibatinib have shown significant advancements in recent years [5]. Pemigatinib received approval from the U.S. Food and Drug Administration (FDA) in April 2020 for the treatment of adults with previously treated, unresectable, locally advanced, or metastatic cholangiocarcinoma with fibroblast growth factor receptor 2 (FGFR2) fusions or rearrangements [6]. However, the therapeutic efficacy of these targeted agents is significantly limited by drug resistance and the short duration of their effectiveness. Currently, there is an absence of reliable biomarkers to predict treatment response or guide therapeutic strategies for pemigatinib-resistant cases. As a result, effective strategies for addressing drug resistance in ICC are urgently needed to facilitate the development of novel therapeutic agents.

β-arrestin 2 (ARRB2), a member of the arrestin/β-arrestin protein family, is implicated in the agonist-mediated desensitization of G protein-coupled receptors (GPCRs), which leads to the selective inhibition of cellular responses to stimuli such as hormones, neurotransmitters, or sensory signals [7–9]. Bile acids, which are chronically elevated in the tumor microenvironment of ICC patients, are potent signaling molecules that act through GPCRs [10]. As a central regulator of GPCR signaling and downstream pathways (including MAPK and Hippo/YAP), ARRB2 serves as a key molecular node that could integrate oncogenic signals from the ICC tumor microenvironment [11, 12]. Furthermore, our prior work demonstrated that prognostically associated long non-coding RNAs promote ICC tumorigenesis by enhancing YAP1 transcription [13]. However, the exact molecular mechanisms through which ARRB2 regulates ICC progression remain largely unexplored, and its role in ICC needs further investigation.

N6-methyladenosine (m6A) is a prevalent and highly conserved messenger RNA (mRNA) epitranscriptomic modification that plays a critical role in gene regulation during post-transcriptional regulation, including RNA splicing, translation, and degradation in eukaryotes. m6A is a dynamic and reversible RNA modification implicated in tumorigenesis, and its function is mediated by m6A-related writers, erasers, and readers [14–16]. Li et al. demonstrated that YTHDF1 promotes the translation of ATG2A and ATG14 by recognizing their m6A sites, thereby modulating cellular autophagy and promoting the malignant progression of hepatocellular carcinoma [17]. However, the potential role and underlying mechanisms of m6A modification in ICC remain largely unexplored.

In this study, we established pemigatinib-resistant ICC cell lines through gradual exposure to pemigatinib and observed a significant upregulation of ARRB2 expression in the resistant RBE and HuCCT1 cells compared to their parental cells. Furthermore, we observed that ARRB2 expression was elevated in ICC patients and correlated with poor prognosis. Both in vitro and in vivo experiments showed that ARRB2 played a critical role in ICC progression. Mechanistically, METTL3 and YTHDF1 were significantly elevated in ICC patients and cells, enhancing ARRB2 translation in an m6A-dependent manner and thereby contributing to pemigatinib resistance. In summary, our study demonstrated that ARRB2 is up-regulated in ICC and plays a crucial role in pemigatinib resistance, suggesting that ARRB2 is a novel biomarker and potential therapeutic target for ICC.

## Materials and methods

### Clinical liver samples

98 pairs of ICC tissue samples were collected from patients undergoing partial hepatectomy. The tissues were preserved in liquid nitrogen for qRT-PCR and Western blot analyses, and fixed in paraformaldehyde for immunohistochemistry (IHC) staining. All patients provided a signed informed consent form. The clinical study was approved by the Ethics Committee of the Third Affiliated Hospital of Sun Yat-sen University and complied with the Declaration of Helsinki.

### In vivo experiments

Nude mice were purchased from Baishitong Biotechnology Co., Ltd (China). Ethical approval for animal experiments was obtained from the South China Agricultural University Animal Ethics Committee (approval NO: RGBIO 2022012501). The experiment on mice was carried out according to the principles and guidelines of animal care and use formulated by the South China Agricultural University (China). Sample sizes were selected empirically, informed by historical data on experimental variability, and were not predetermined using formal statistical power calculations. No mice were excluded from the quantifications, and no randomization method was used to divide the mice. For orthotopic implantation, 1 × 10^7^ RBE cells were injected into the left lobes of the livers of 8-week-old male nude mice. Livers were harvested 4 weeks after injection for the analysis of ICC tumorigenesis. For the NICD/AKT-induced ICC model, hydrodynamic injection was used to induce ICC. Twenty micrograms of NICD, 4 μg AKT, and 1 μg sleeping beauty 100 (SB100) transposase plasmids (Miaolingbio, China) were diluted in 2 ml 0.9% NaCl, sterile filtered, and injected into a lateral tail vein within 5–7 s [18].

### AAV8 injection

AAV8 was used to construct short hairpin RNAs (shRNAs) targeting ARRB2 in the liver (GeneChem, China). Recombinant virus (1.5 × 10^11 ^v.g) was diluted in 0.2 mL of a saline solution and injected into the tail veins of the mice. The NICD/AKT-induced ICC model was established 2 weeks following AAV8 injection.

### Cell culture

The human ICC cell lines RBE and HuCCT1 were obtained from American Type Culture Collection (ATCC). RBE and HuCCT1 cells were cultured in Dulbecco’s modified Eagle’s medium (DMEM, Gibco) supplemented with 10% fetal bovine serum (CellCook, China) and 1% Penicillin-Streptomycin (Gibco, USA). Cells were grown in a 5% CO_2_ cell culture incubator (Thermo Scientific, USA) at 37 °C. For drug administration, RBE and HuCCT1 cells were treated with Pemigatinib (10 μM, Selleck Chemicals, USA) for 72 h or YAP-specific inhibitor Verteporfin (1 μM, Selleck Chemicals) for 24 h or c-Raf-specific inhibitor GW5074 (1 μM, Selleck Chemicals) for 24 h. ShRNA, siRNA and plasmid were used to modulate the expression of ARRB2. ARRB2-shRNA plasmid (GeneCopoeia, USA), ARRB2-siRNA (RiboBio, China) and ARRB2-overexpressing plasmid (GeneCopoeia) were transfected using Lipofectamine 3000 (Invitrogen, USA) according to the manufacturer’s instructions. All transfections were independently performed at least three times. All cell lines were authenticated using short tandem repeat (STR) profiling and routinely monitored for mycoplasma contamination and microbial infections through bi-monthly PCR-based testing and microbiological culture assays, with initial authentication performed upon acquisition and subsequent checks conducted every six months throughout the study period.

### Establishment of pemigatinib-resistant cell lines

RBE and HuCCT1 cells were treated with increasing doses of pemigatinib. After 72 h, Cell Counting Kit-8 (NCM Biotech, China) was used to determine cell viability at 450 nm after incubating at 37 °C for an additional 2 h. Then, the half-maximal inhibitory concentration (IC50) of RBE and HuCCT1 cells to pemigatinib was further calculated. Then, we incubated RBE and HuCCT1 cells with pemigatinib at concentrations just below their IC50. The dose of pemigatinib was slowly increased to 10 μM. After several months, we successfully established the pemigatinib-resistant cells, termed RBE-PR and HuCCT1-PR. After establishment, the resistant cell line continued to be cultured in 10 μM pemigatinib.

### Long-term clonogenic assays

Cells were planted in 6-well plates at a density of 800 cells per well and then treated with 10 μM pemigatinib for an additional 7 days (medium was changed twice a week). Visible colonies were fixed with 4% paraformaldehyde and stained with 0.1% crystal violet to calculate colony area. Clone numbers in each well were quantified using ImageJ software.

### Live/dead cell staining

The live–dead cell staining kit (Beyotime Biotechnology, China) was performed according to the manufacturer’s instructions. Briefly, cells were cultured in 12-well plates with a density of 5 × 10^4^ cells per well. Propidium iodide and calcium-AM were diluted to final concentrations of 5 μM and 2 μM in PBS, respectively. After 4 days, 100 μl of mixed solution was added to each well, and the cells were stained at 37 °C for 15 min.

### Immunofluorescence (IF) staining

The cells were fixed with 4% paraformaldehyde for 20 min at room temperature, followed by incubation with 0.5% Triton (Sigma‒Aldrich, USA) at room temperature for 10 min to increase cell membrane permeability. Then, the cells were blocked with 10% bull serum albumin (BSA, Sigma‒Aldrich) for 1 h and treated with ARRB2 (1:200 dilution, Cell Signaling Technology, USA) diluted in 1% BSA overnight at 4 °C. Finally, the cells were washed with PBS and incubated with secondary antibody [Cy3-labeled Goat Anti-Rabbit IgG (Beyotime)] for 1 h at 37 °C in the dark, and nuclear staining was performed with 4′,6-diamidino-2-phenylindole (DAPI, KeyGen, China).

### IHC and immunofluorescence staining

Liver tissues were fixed in 10% paraformaldehyde, embedded in paraffin and then cut into sections of 4 mm thickness. After dewaxing, hydrating, and antigen repair, the sections were incubated with corresponding primary antibodies overnight at 4 °C, including ARRB2, METTL3 and YTHDF1 (1:200 dilution, Cell Signaling Technology, USA). After washing three times with 1× phosphate buffer saline (PBS), the sections were incubated with horseradish peroxidase (HRP)-linked secondary antibody (DAKO, Denmark) for 1 h at room temperature. At last, the sections were visualized using DAB (DAKO) and counterstained using hematoxylin. IHC scoring was performed by two blinded pathologists with expertise in ICC diagnosis, using a 0–15 point relative scoring scheme.

After dewaxing, hydrating, and antigen repairing, the sections were washed three times with 1× PBS and then permeabilized and blocked in 1× PBS with 0.2% Triton X-100 and 5% normal goat serum, followed by incubation with primary antibodies ARRB2 and YAP (1:200 dilution, Cell Signaling Technology) overnight at 4 °C. The sections were washed and then incubated with FITC-labeled Goat Anti-Rabbit IgG and Cy3-labeled Goat Anti-Mouse IgG (1:500 dilution, Beyotime Biotechnology) for 1 h at room temperature, followed by subsequent washes. Additional staining with DAPI was used to label nuclei.

### Western blot

Western blot analysis of liver tissue was performed according to the manufacturer’s instructions (Beyotime). Equal concentrations of protein from liver tissue were separated by 12.5% sodium dodecyl sulfate-polyacrylamide (SDS-PAGE) gel electrophoresis and transferred to polyvinylidene fluoride (PVDF) membranes (Millipore, USA). Subsequently, the membranes were incubated overnight at 4 °C with specific antibodies against the following proteins: ARRB2 (Cat# 3857), METTL3 (Cat# 86132), YTHDF1 (Cat# 57530), YAP (Cat# 14074), Phospho-YAP (Cat# 13008, p-YAP), LATS1 (Cat# 3477), Histone-3 (Cat# 9715), c-Raf (Cat# 53745), Phospho-c-Raf (Cat# 9427, p-c-Raf), MEK1/2 (Cat# 8727), Phospho-MEK1/2 (Cat# 9154), ERK1/2 (Cat# 4695), Phospho-MEK1/2 (Cat# 9101) and GAPDH (Cat# 2118, 1:1000 dilution, Cell Signaling Technology). The next day, membranes were incubated with a horseradish peroxidase-conjugated anti-rabbit or mouse antibody at room temperature. The signal detection and result record were finished by Tanon-5200CE Chemiluminescent Imaging System (Tanon Science and Technology, China).

### RNA isolation and quantitative real-time reverse transcription-PCR

Total RNA was extracted using Trizol (Roche, Switzerland). Reverse transcription of total RNA (1.5 μg) with a Revert Aid First Strand cDNA synthesis kit (Roche) according to the manufacturer’s instructions. qPCR was performed using the SYBR Green I master kit (Roche) on LightCycler 480 (Roche). Data were analyzed using the 2^–ΔΔCt^ method, and β-actin RNA was used as an endogenous control. Primers for ARRB2 are as follows: forward: 5′- AGAAAAACCCGGGACCAG -3′, and reverse: 5′- GATCCCCAGCACCTCCTT -3′. Primers for AKR1C1 are as follows: forward: 5′-AAA GCCAGGTGAGGAAGTGA -3′, and reverse: 5′-CATGTGGCACAGAGATCCAC -3′. Primers for ISG20 are as follows: forward: 5′- ACACGTCCACTGACAGGCTGTT-3′, and reverse: 5′- ATCTTCCACCGAGCTGTGTCCA-3′. Primers for OASL are as follows: forward: 5′- TTCAGCGAGCTGCAGAGAAA -3′, and reverse: 5′- CCCTCTGCTCCACTGTCAAG -3′. Primers for PSMB9 are as follows: forward: 5′- TGCTGACTCGACAGCCTTTT-3′, and reverse: 5′- GCCCAAGATGACTCGATGGT-3′. Primers for β-actin are as follows: forward: 5′- CATGTACGTTGCTATCCAGGC -3′, and reverse: 5′- CTCCTTAATGTCACGCACGAT -3′, which were used as a reference for normalization.

### mRNA stability assays

The indicated cells were cultured in 12-well plates, followed by si-METTL3 or si-YTHDF1 for 24 h. Then, actinomycin D (MCE, China) was added to each well at a final concentration of 5 μg/ml, and the cells were collected after 0, 1, 2, 6, 12, and 24 h of incubation. Total RNA was isolated and subsequently subjected to qRT‒PCR to quantify the relative abundance of ARRB2 mRNA (relative to 0 h).

### RNA sequencing

Total RNA was extracted using the Trizol reagent kit (Invitrogen) according to the manufacturer’s protocol. RNA quality was assessed on an Agilent 2100 Bioanalyzer (Agilent Technologies, USA) and checked using RNase-free agarose gel electrophoresis. After total RNA was extracted, eukaryotic mRNA was enriched by Oligo(dT) beads. Then the enriched mRNA was fragmented into short fragments using the fragmentation buffer and reverse transcribed into cDNA by using NEBNext Ultra RNA Library Prep Kit for Illumina (New England Biolabs, USA). The purified double-stranded cDNA fragments were end-repaired, a base added, and ligated to Illumina sequencing adapters. The ligation reaction was purified with the AMPure XP Beads (1.0X). Ligated fragments were subjected to size selection by agarose gel electrophoresis and polymerase chain reaction (PCR) amplification. The resulting cDNA library was sequenced using Illumina Novaseq6000 by Gene Denovo Biotechnology Co. (China).

### RNA m6A dot-blot assay

Total RNA was extracted from cells using TRIzol reagent (Invitrogen), and then diluted with nuclease-free water to 500 ng and subsequently denatured at 95 °C for 5 min. The 500/250/100 ng mRNA samples were spotted onto a Hybond-N+ membrane (GE Healthcare, USA) and then crosslinked to the membrane by two rounds of 2400 UV crosslinking (5 min each time). Afterward, the membrane was washed with 0.1% TBST (compounded by DEPC water), blocked with 5% nonfat milk in 0.1% TBST, and incubated with an anti-m6A antibody (Cat# 56593, 1:1000, Cell Signaling Technology) overnight at 4 °C. After three washes with PBST, the blotting was incubated with horseradish peroxidase-conjugated anti-rabbit immunoglobulin G (Cat# 7074, 1:1000, Cell Signaling Technology) for 1 h at room temperature with gentle shaking. After rinsing, membranes were developed with ECL Western Blotting Detection Kit (Advansta, USA). The same amount of mRNA was spotted on the membrane, stained with 0.02% methylene blue in 0.3 M sodium acetate (pH 5.2) for 10 min, and rinsed with PBST three times for 5 min each as the loading control.

### RNA immunoprecipitation (RIP)

The ICC cells were harvested on ice using IP lysis buffer (Beyotime) supplemented with RNase Inhibitor and Protease Inhibitor Cocktail (MCE). Add YTHDF1 antibody (Cell Signaling Technology) to pre-washed Protein A/G magnetic beads (Thermo Scientific) and incubate with rotation for 2 h at room temperature. And the samples were subsequently incubated with magnetic beads conjugated to YTHDF1 antibody at 4 °C overnight. The Co-immunoprecipitated RNAs were eluted using TRNzol reagent (Thermo Scientific), and the relative enrichment of YTHDF1 mRNA was detected by RT-qPCR. GAPDH was used as a negative control.

### RNA pull-down assay

Pull-down assay was carried out with Pierce Magnetic RNA pull-down Kit (Thermo Scientific) following the manufacturer’s protocol. Samples were UV-crosslinked (254 nm) with a Stratalinker ultraviolet cross-linker for 10 min. The eluted proteins from the RNA pull-down assay were resolved by SDS-PAGE and silver-stained with the Pierce Silver Stain for Mass Spectrometry (Thermo Scientific). The precipitated products were analyzed by liquid chromatography tandem mass spectrometry (LC-MS, The Thermo Q Exactive HF-X mass spectrometer, Thermo Scientific) or western blot.

### m6A MeRIP-qPCR

MeRIP assays were performed following the manufacturer’s instructions (IEMed MeRIP m6A Kit, China). Briefly, RNA from ICC cells was isolated and fragmented into products that were 300 bp or fewer nucleotides in size at 95 °C for 90 min. Magnetic beads A/G were incubated with anti-m6A antibody for 30 min at room temperature. Then, the anti-m6A antibody-bound magnetic beads were washed, added to DNA-free RNA and incubated for 1 h at room temperature. The beads were then washed, and the bound RNA was eluted. Input and RIP samples were finally purified using the miRNeasy Mini Kit (QIAGEN, Germany). Further m6A enrichment was analyzed using qRT‒PCR, and the results were normalized by the input.

### Surface plasmon resonance (SPR) assay

The SPR assays were performed to test the interactions between FGFR2 and Pemigatinib using a Biacore 1 K^+^ (GE Healthcare) with a CM5 sensor chip (GE Healthcare) at 25 °C. The buffer system was PBST (10 mM Na_2_HPO, 2 mM KH_2_PO_4_, pH 7.4, 137 mM NaCl, 2.7 mM KCl and 0.05% Tween 20). FGFR2 was pre-immobilized at 1900RU on the CM5 sensor chip using standard amine coupling chemistry with a 40 μg/mL concentration. Serially diluted Pemigatinib (0.78125 nM, 3.125 nM, 6.25 nM, 12.5 nM and 50 nM) was flowed over the chip to evaluate FGFR2 binding. The equilibrium dissociation constants (KD) of the FGFR2 interaction were analyzed using a 1:1 binding model and a steady-state affinity model in the Biacore T200 Evaluation Software (GE Healthcare), respectively.

### Statistical analysis

A minimum of three biological replicates was employed for all experimental procedures. Qualitative data were obtained from at least three independent replicates. Quantitative data are expressed as mean ± standard deviation. Prior to employing *t*-tests (for comparing two groups) and ANOVA (for comparing multiple groups), normality was assessed using the Shapiro–Wilk test. Additionally, the homogeneity of variances was checked with Levene’s test. Relationship analyses were performed using a linear regression model. For multivariate regression models, all covariates with *P* ≤ 0.05 in the univariate analysis were included in the multivariate analysis. A *P*-value < 0.05 was considered statistically significant. SPSS software (version 25.0; IBM, USA) was used for all analyses.

## Results

### ARRB2 is up-regulated in pemigatinib-resistant intrahepatic Cholangiocarcinoma cells

**Fig. 1:**
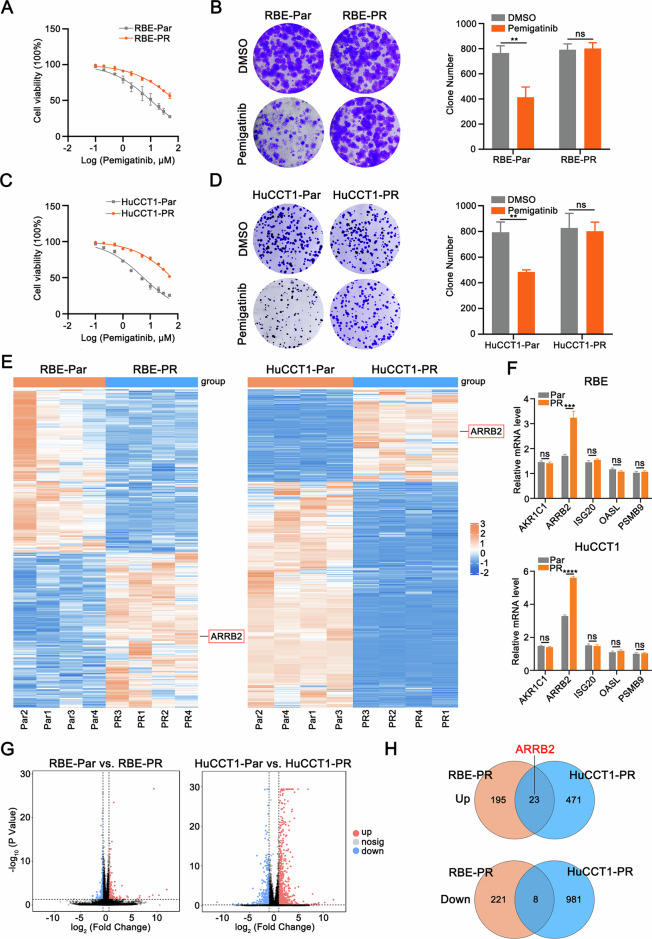
ARRB2 is up-regulated in pemigatinib-resistant intrahepatic Cholangiocarcinoma cells. **A** Half maximal inhibitory pemigatinib concentration curves of RBE-Par cells and RBE-PR cells. **B** Colony formation (left) and quantification (right) of the RBE and RBE-PR after pemigatinib treatment for 10 days. **C** Half maximal inhibitory pemigatinib concentration curves of HuCCT1-Par cells and HuCCT1-PR cells. **D** Colony formation (left) and quantification (right) of the HuCCT1 and HuCCT1-PR after pemigatinib treatment for 10 days. **E** Heatmap showing RNA differential expression of genes between RBE-Par and RBE-PR cells (left), and HuCCT1-Par and HuCCT1-PR cells (right). **F** Real-time PCR analysis of the expression of the top 5 genes that are commonly up-regulated in both data sets in RBE (up) and HuCCT1 (down) cells. **G** Volcano plot showing differential gene expression between RBE-Par and RBE-PR cells (left), and HuCCT1-Par and HuCCT1-PR cells (right). **H** Venn diagram analysis. The RBE-PR cells exhibited 195 up-regulated genes compared with RBE-Par cells. The HuCCT1-PR exhibited 471 up-regulated genes compared with HuCCT1-Par cells; 23 genes that are commonly up-regulated in both data sets. All results are presented as mean ± SD, and statistical significance was assessed using a 2-tailed Student *t* test. **P < 0.01, ***P < 0.001, and ****P < 0.0001. ns not significant, Par parental, PR pemigatinib resistant.

To investigate the molecular mechanisms underlying resistance to Pemigatinib in ICC, we first established Pemigatinib-resistant RBE cells by gradually exposing the cells to increasing concentrations of Pemigatinib. These resistant cells exhibited elevated IC50 values for Pemigatinib (Fig. 1A) and demonstrated greater variability in cellular responses to Pemigatinib treatment (Fig. 1B). To further investigate the integrity of the resistance model, we performed binding assays between Pemigatinib and FGFR2 protein purified from Pemigatinib-resistant RBE cells. Surface plasmon resonance (SPR) analysis demonstrated a high binding affinity between Pemigatinib and FGFR2, with a dissociation constant (KD) of 1.13 × 10⁻⁸ M (Supplementary Fig. 1A, B). Using the same methodology, we also generated Pemigatinib-resistant HuCCT1 cells (Fig. 1C, D). To identify potential molecular targets associated with Pemigatinib resistance in ICC, we conducted mRNA profiling on both cell lines (Fig. 1E). In parallel, qRT-PCR validation confirmed the reliability of the transcriptome sequencing results (Fig. 1F). The volcano plots, which highlight the differential expression of candidate genes across the entire transcriptome, are shown in Fig. 1G. Transcriptomic analysis showed that expression levels of 23 genes were commonly increased (>2-fold, Fig. 1H), among which ARRB2 was the top up-regulated gene.

### ARRB2 regulated the sensitivity of ICC cells to pemigatinib

**Fig. 2:**
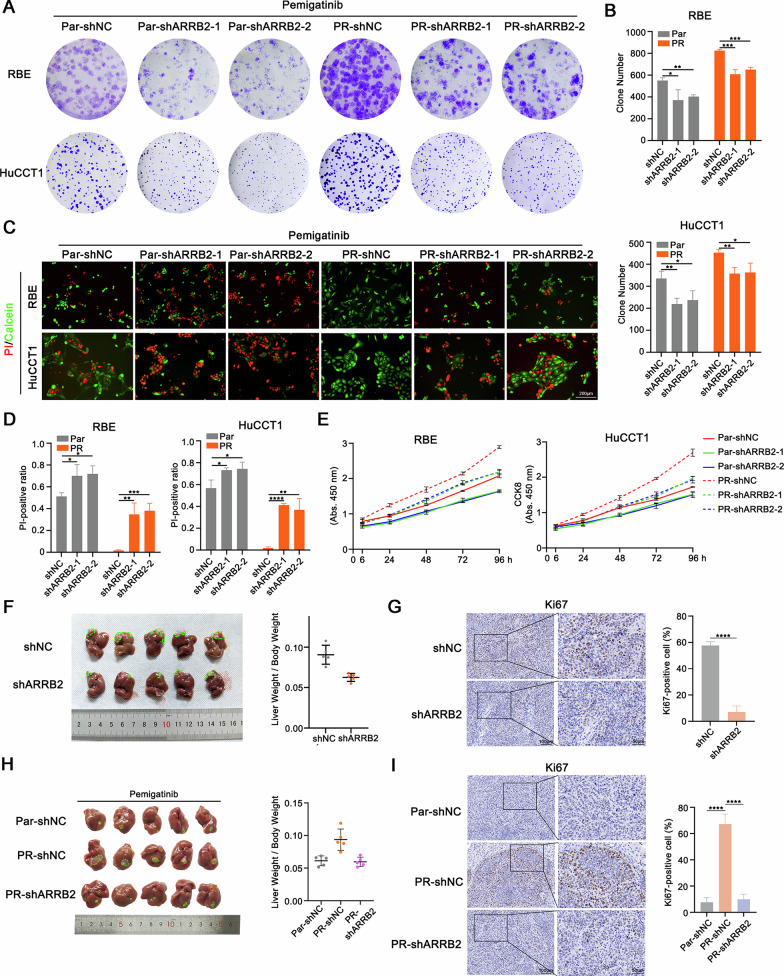
ARRB2 regulated the sensitivity of ICC cells to pemigatinib. **A** Colony formation and quantification (**B**) analysis of ARRB2 knockdown in RBE (up) and HuCCT1 (down) cells. **C** Representative live/dead cell staining images and quantification (**D**) analysis of ARRB2 knockdown in RBE (left) and HuCCT1 (right) cells. **E** Growth curve analysis of RBE (up) and HuCCT1 cells (down) upon ARRB2 knockdown. **F** Representative images of livers in the xenograft ICC model (left) and quantification of liver weight/body weight (right, *n* = 5/group). **G** Representative images (left) and quantification (right) of immunohistochemical (IHC) staining of Ki67 in xenograft model. (**H**) Nude mice were orthotopically xenografted with RBE cells and orally with pemigatinib (1 mg/kg) daily. Representative images (left) and liver weight/body weight (right) of mice in the indicated groups are shown (*n* = 5/group). **I** Representative images (left) and quantification (right) of IHC staining of Ki67 in the xenograft model. All results are presented as mean ± SD, and statistical significance was assessed using a 2-tailed Student *t* test. *P < 0.05, **P < 0.01, ***P < 0.001, and ****P < 0.0001. Par parental, PR pemigatinib resistant.

To further elucidate the role of ARRB2 in regulating pemigatinib resistance, we knocked down ARRB2 in the acquired resistant RBE-PR and HuCCT1-PR cell lines using two independent short hairpin RNAs. Pemigatinib treatment alone had no significant effect on cell proliferation or apoptosis in these cells. However, ARRB2 knockdown rendered the cells more sensitive to pemigatinib treatment, as evidenced by reduced colony formation (Fig. 2A, B), increased cell survival (Fig. 2C, D), and impaired cell growth curve (Fig. 2E). In vivo xenograft with stable ARRB2-silenced RBE cells further demonstrated that ARRB2 is essential for ICC proliferation (Fig. 2F). This finding was corroborated by IHC analysis, which showed a marked reduction in the Ki-67 positive cell ratio upon ARRB2 knockdown (Fig. 2G). Additionally, pemigatinib was applied to an orthotopic ICC model established with RBE-PR cells, with or without ARRB2 knockdown. ARRB2 knockdown almost completely eradicated ICC tumors, exhibited much higher antitumor efficacy than pemigatinib treatment (Fig. 2H, I). These findings demonstrate that ARRB2 plays a critical role in mediating pemigatinib resistance in ICC cells.

### ARRB2 Correlates with clinical staging and prognosis in ICC patients

**Fig. 3:**
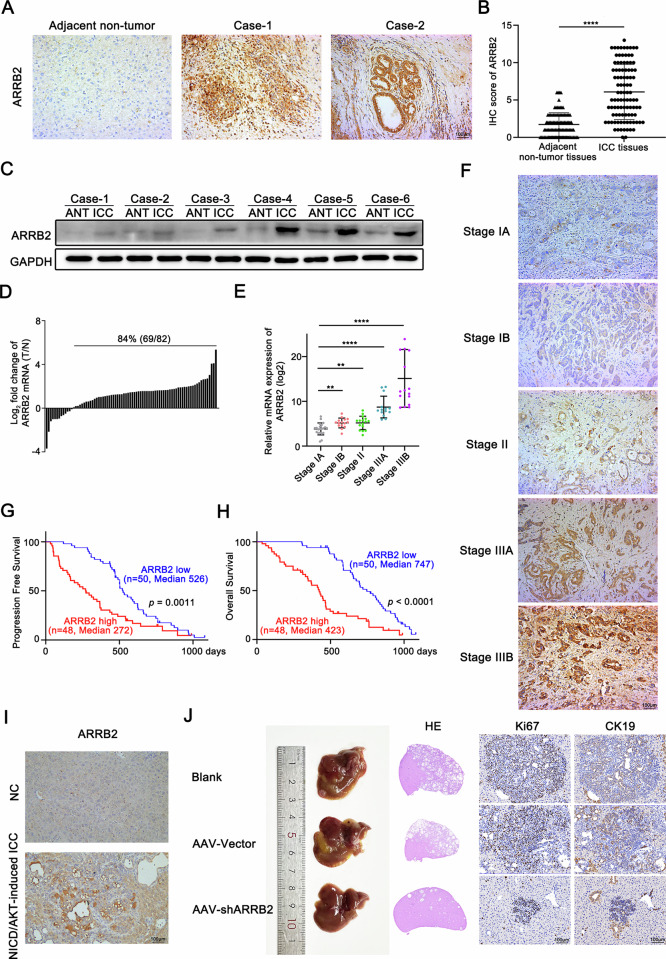
ARRB2 is up-regulated and predicts poor prognosis in patients with intrahepatic Cholangiocarcinoma. **A** Representative images of immunohistochemical (IHC) staining of ARRB2 in ICC patients (*n* = 98). **B** Quantification of IHC staining of ARRB2 in ICC patients (*n* = 98). **C** The expression of ARRB2 was determined by western blot analysis in 6 pairs of ICC tissues and adjacent normal tissues (*n* = 6). **D** The mRNAs of ARRB2 in 82 pairs of ICC tissues and adjacent normal tissues were detected with qPCR (*n* = 82/group). **E** The mRNAs of ARRB2 in different pathological stages ICC tissues were detected with qPCR (*n* = 21 for stage IA, 15 for stage IB, 16 for stage II, 14 for stage IIIA, 16 for stage IIIB). **F** Quantification of IHC staining of ARRB2 in different pathological stages in ICC patients (*n* = 98). Kaplan–Meier analysis for progression-free survival (**G**) and overall survival (**H**) was performed according to ARRB2 levels of IHC staining in ICC patients (*n* = 98). **I** Representative images of IHC staining of ARRB2 in the NICD/AKT-induced ICC model. **J** Representative images of livers in the NICD/AKT-induced ICC model (left) hematoxylin and eosin (H&E) staining (middle) and IHC staining of Ki67 and CK19 (right, *n* = 5/group). All results are presented as mean ± SD, and statistical significance was assessed using a 2-tailed Student *t* test. **P < 0.01 and ****P < 0.0001.

To assess the clinical significance of ARRB2 in ICC, we analyzed its expression in clinical specimens from ICC patients. Univariate and multivariate analysis demonstrated that elevated ARRB2 expression in ICC tissues was significantly associated with more aggressive clinical features (Supplementary Table 1). Furthermore, we examined ARRB2 expression in 98 tumor-adjacent tissues and ICCs using an IHC assay (Fig. 3A). The IHC scores for ARRB2 were significantly higher in ICCs compared to adjacent non-tumor tissues (Fig. 3B). ARRB2 protein expression was further quantified by western blot (WB) analysis (Fig. 3C) and qRT-PCR (Fig. 3D), both of which produced consistent results. Additionally, we investigated the expression levels of ARRB2 across different pathological stages in 82 ICCs according to the AJCC staging system. Analysis revealed significant differences in ARRB2 expression levels between major pathological stages (Fig. 3E, F). Kaplan–Meier survival analysis further indicated that high ARRB2 expression in ICCs correlated with poorer progression-free survival and overall survival (Fig. 3G, H).

Based on the results described above, a role for ARRB2 in the progression of ICC was further investigated using a recently described ICC model driven by oncogenes AKT and active form of Notch, NICD. The result revealed elevated ARRB2 expression in NICD/AKT-induced ICC tissues compared to normal liver tissues (Fig. 3I and Supplementary Fig. 2A). What’s more, AAV8-mediated knockdown of hepatic ARRB2 significantly suppressed tumor growth, as evidenced by reduced tumor number and size. IHC analysis demonstrated that ARRB2 knockdown led to a corresponding decrease in the proportion of Ki67- and CK19-positive cells (Fig. 3J and Supplementary Fig. 2B, C). Collectively, these findings suggest that ARRB2 upregulation in ICC not only diminishes pemigatinib sensitivity but also serves as a significant prognostic factor associated with poor outcomes in ICC patients.

### METTL3 augments the expression of ARRB2 mRNA via m6A methylation

**Fig. 4:**
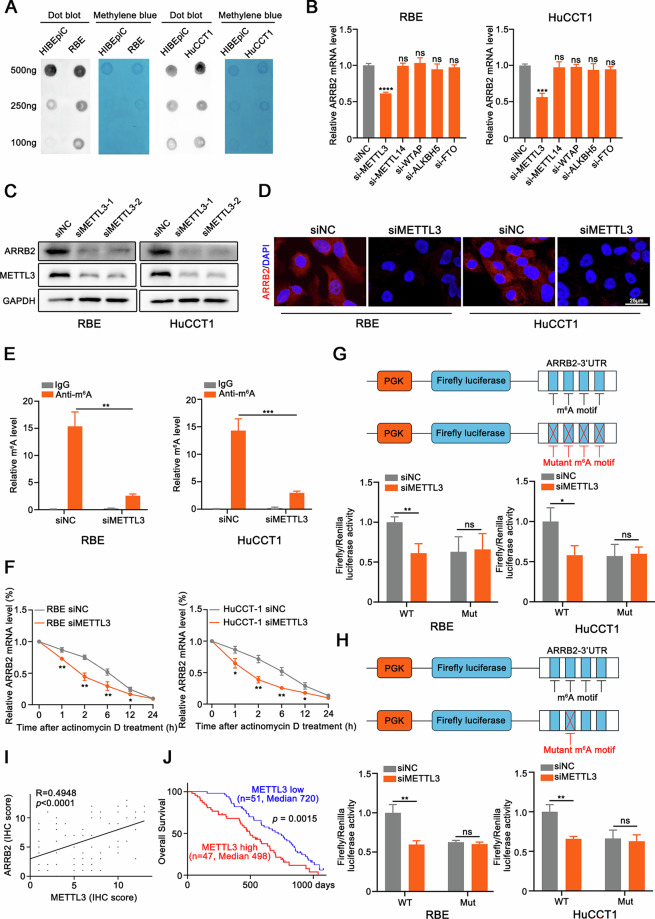
METTL3-mediated N6-methyladenosine induces the upregulation of ARRB2 and predicts poor prognosis in patients with intrahepatic Cholangiocarcinoma. **A** Dot blot analysis of m6A modification in HIBEpiC, RBE and HuCCT1 cells. **B** ICC cells were transfected with siMETTL3, siMETTL14, siWTAP, siALKBH5, siFTO or siNC for 24 h followed by real-time PCR analysis. **C** The expression of ARRB2 was determined by western blot analysis in ICC cells that were transfected with siMETTL3. **D** Immunofluorescence staining of ARRB2 (red fluorescence) in ICC cells transfected with siMETTL3 or siNC. Nuclei were counterstained with DAPI (blue fluorescence). **E** PCR analysis of immunoprecipitation of m6A-modified RNA in ICC cells transfected with siMETTL3 or siNC. **F** Stability of ARRB2 mRNA was measured after Actinomycin D treatment. **G**, **H** Relative luciferase activity of pmirGLO-hARRB2-3’ UTR with either wild-type or mutant (A-to-T mutation) m6A sites in ICC cells co-transfected with siMETTL3 or siNC, respectively. Firefly luciferase activity was measured and normalized to Renilla luciferase activity. **I** The correlation between the level of ARRB2 and METTL3 in ICC patients (*n* = 98) was determined by IHC score. **J** Kaplan–Meier analysis for overall survival was performed according to METTL3 levels of IHC staining in ICC patients (*n* = 98). All results are presented as mean ± SD and statistical significance was assessed using a 2-tailed Student *t* test. *P < 0.05, **P < 0.01, and ***P < 0.001. ns not significant.

The levels of m6A methylation were investigated by dot-blotting using an anti-m6A antibody, and we observed a significant upregulation in m6A levels in RBE and HuCCT1 compared to HIBEpiC (Fig. 4A). To further investigate the mechanisms underlying m6A hypermethylation in ICC cells, we knocked down a series of m6A methyltransferases and found that ARRB2 mRNA expression was substantially decreased following METTL3 knockout (Fig. 4B–D). Methylated RNA immunoprecipitation quantitative PCR data indicated that the m6A-specific antibody significantly reduced the enrichment of ARRB2 mRNA following METTL3 knockdown (Fig. 4E). Treatment of ICC cells with actinomycin D suggested that METTL3 regulates the stability of ARRB2 mRNA (Fig. 4F). The SRAMP website (http://www.cuilab.cn/sramp/) was employed to predict the mRNA sequence of ARRB2 to pinpoint the m6A site influenced by METTL3. The prediction results exhibited four potential m6A modification sites with very high confidence in the mRNA sequence of ARRB2 (Supplementary Fig. 3A).

To further elucidate the effect of m6A modification on ARRB2 mRNA stability, we performed luciferase reporter and mutagenesis assays. As expected, METTL3 knockdown decreased luciferase activity in the wild-type group but not in the mutant group, further supporting the role of m6A modification in ARRB2 mRNA stability (Fig. 4G). Furthermore, we have performed comprehensive site-directed mutagenesis experiments to validate the exact functional m6A modification site(s) on ARRB2 mRNA. The results demonstrate that mutation of a specific site, which we have identified as the adenosine at position 278 (A278) within the ARRB2 transcript, significantly abolished the METTL3-mediated degradation of luciferase activity. In contrast, mutations at other candidate sites had minimal effects (Fig. 4H and Supplementary Fig. 3B–D). Notably, a correlation between ARRB2 expression and METTL3 levels was observed via IHC in 98 tumor-adjacent tissues and ICCs (Fig. 4I). Moreover, our analysis revealed that elevated METTL3 expression was associated with poorer prognosis in ICC patients (Fig. 4J).

### M6A reader YTHDF1 enhances the stability of ARRB2 in ICC

**Fig. 5:**
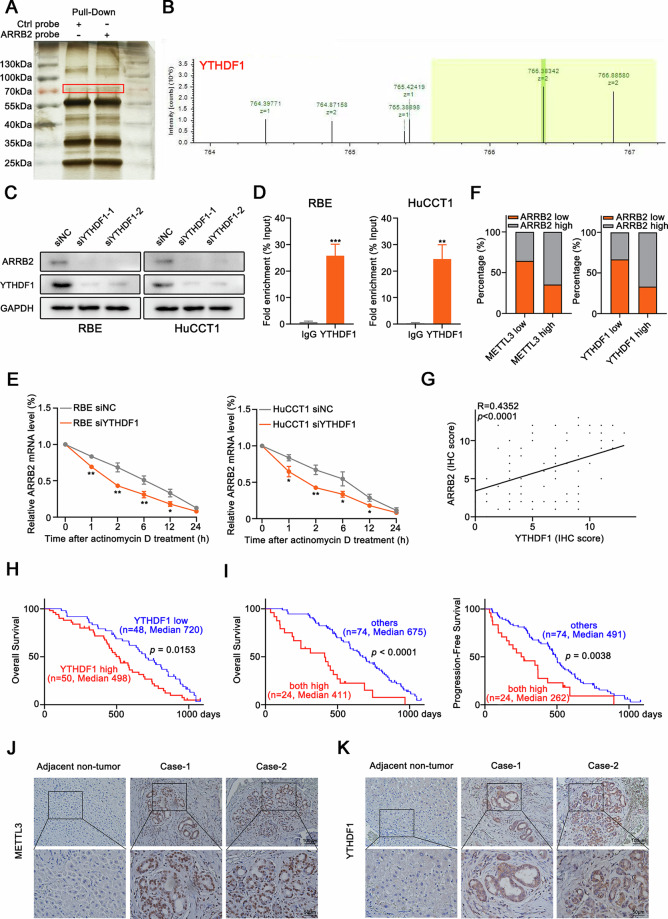
METTL3-mediated N6-methyladenosine modification of ARRB2 messenger RNA maintains its YTHDF1-dependent stability. **A** RNA pull-down assays revealed that the band at about 70 kDa was presented in the complex pull-down by ARRB2. **B** Mass spectrometry analysis bands were cut from the SDS-PAGE and analyzed via mass spectrometry. **C** The expression of ARRB2 was determined by western blot analysis in ICC cells were transfected with siYTHDF1. **D** PCR analysis of immunoprecipitation of m6A-modified RNA in ICC cells transfected with siYTHDF1 or siNC. **E** Stability of ARRB2 mRNA was measured after Actinomycin D treatment. **F** The correlation between ARRB2 levels and METTL3 or YTHDF1 expression of IHC staining in ICC patients (*n* = 98) was evaluated using a χ^2^ test. **G** The correlation between the level of ARRB2 and YTHDF1 in ICC patients (*n* = 98) was determined by IHC score. **H** Kaplan–Meier analysis for overall survival was performed according to YTHDF1 levels of IHC staining in ICC patients (*n* = 98). **I** Overall survival and progression-free survival were compared between the patients exhibiting high ARRB2, high METTL3, and high YTHDF1 levels and the other patients in ICC patients (*n* = 98) using Kaplan–Meier analysis. **J** Representative images of IHC staining of METTL3 in ICC patients (*n* = 98). (**K**) Representative images of IHC staining of YTHDF1 in ICC patients (*n* = 98). All results are presented as mean ± SD, and statistical significance was assessed using a 2-tailed Student *t* test. *P < 0.05, **P < 0.01, and ***P < 0.001.

A recent study reported that IGF2BP1/2/3 and YTHDF1/2/3 constitute a distinct family of m6A readers that target thousands of mRNA transcripts through the recognition of the m6A motif [16]. Therefore, we explored the effect of m6A readers on ARRB2 mRNA stabilization. PRIdictor (Protein–RNA Interaction Predictor) is a tool used to predict mutual binding sites in RNA and protein at the nucleotide or residue level (http://bclab.inha.ac.kr/pridictor). PRIdictor analyses indicate that YTHDF1 strongly associates with ARRB2 mRNA (Supplementary Fig. 4A–G), with the binding area being coherent with the distribution of ARRB2 mRNA m6A modification motifs (Supplementary Fig. 3A). RNA pull-down assays combined with mass spectrometry suggested that YTHDF1 is a potential ARRB2-interacting protein (Fig. 5A, B), and western blotting confirmed that YTHDF1 could pull down ARRB2 (Supplementary Fig. 4H). Knockdown of YTHDF1 markedly suppressed ARRB2 mRNA expression (Fig. 5C). RNA binding protein immunoprecipitation assays suggested the recognition and binding of YTHDF1 with ARRB2 mRNA in RBE and HuCCT1 (Fig. 5D). Furthermore, the stability of ARRB2 mRNA was found to be less stable upon YTHDF1 knockdown (Fig. 5E). Expression of METTL3 or YTHDF1 was strongly correlated with ARRB2 in ICC tissues (Fig. 5F). A significant correlation between ARRB2 expression and YTHDF1 levels was observed via IHC in 98 tumor-adjacent tissues and ICCs (Fig. 5G), and elevated YTHDF1 expression was associated with poorer prognosis in ICC patients (Fig. 5H). Additionally, our analysis revealed that elevated expression of ARRB2, METTL3, and YTHDF1 was associated with a poorer prognosis in ICC patients (Fig. 5I). These results were further confirmed by METTL3 and YTHDF1 IHC staining (Fig. 5J, K). In summary, we have elucidated the critical role of the YTHDF1-METTL3-ARRB2 axis in the malignant progression of ICC and survival prognosis in ICC patients.

### MAPK and Hippo signaling pathways determine the pemigatinib sensitivity in intrahepatic Cholangiocarcinoma

**Fig. 6:**
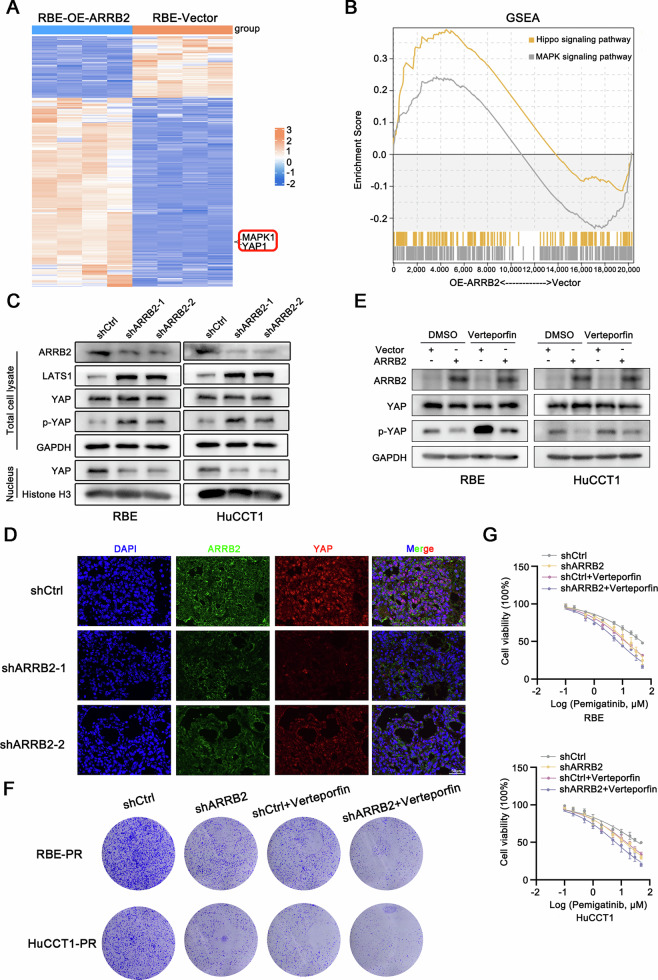
Hippo signaling pathways determine pemigatinib response in intrahepatic Cholangiocarcinoma. **A** Heatmap showing RNA differential expression of genes between ARRB2 overexpression and control RBE cells. **B** Gene set enrichment analysis shows the enrichment of gene sets positively related to MAPK and Hippo pathway in ARRB2 overexpression or control RBE cells. **C** The expressions of ARRB2, LATS1, YAP and p-YAP were determined by western blot analysis in ICC cells that were transfected with shARRB2. **D** Dual-color immunofluorescence for ARRB2 (green fluorescence) and YAP (red fluorescence) in xenograft model. Nuclei were counterstained with DAPI (blue fluorescence). **E** The expressions of ARRB2, YAP and p-YAP were determined by western blot analysis in ICC cells that were transfected with an ARRB2-overexpressing plasmid following Verteporfin treatment. **F** Colony formation in ARRB2-knockdown RBE-PR (up) and HuCCT1-PR (down) cells following Verteporfin treatment. **G** Half maximal inhibitory pemigatinib concentration curves of ARRB2-knockdown RBE-PR (up) and HuCCT1-PR (down) cells following Verteporfin treatment. PR pemigatinib resistant.

To elucidate the mechanism underlying the promotion of ICC by ARRB2, we profiled gene expression in ARRB2-overexpressing and control RBE cells using RNA sequencing. The RNA sequencing data revealed significant changes in the expression of genes related to MAPK and Hippo signaling (Fig. 6A). KEGG signaling pathway enrichment analysis showed that MAPK and Hippo signaling pathways were the top-enriched pathways in ARRB2-overexpressing RBE cells (Fig. 6B). Previous studies have shown that the MAPK and Hippo signaling pathways play crucial roles in the proliferation of ICC. We then investigated the regulation of the MAPK and Hippo signaling pathways by ARRB2. YAP activity is controlled by LATS kinases, which are known to be crucial for upstream YAP inactivation. We conducted ARRB2 knockdown in both RBE and HuCCT1 cell lines. Western blot analysis demonstrated that ARRB2 suppression enhanced LATS kinase-dependent phosphorylation of YAP and reduced YAP-mediated transcriptional activity (Fig. 6C). To directly confirm YAP nuclear translocation, immunofluorescence analysis revealed that YAP nuclear accumulation was significantly attenuated upon ARRB2 knockdown (Fig. 6D). Furthermore, treatment with the YAP-specific inhibitor Verteporfin not only reduced YAP nuclear accumulation (Fig. 6E) but also further enhanced the sensitivity of ARRB2-knockdown resistant cells to pemigatinib (Fig. 6F, G).

**Fig. 7:**
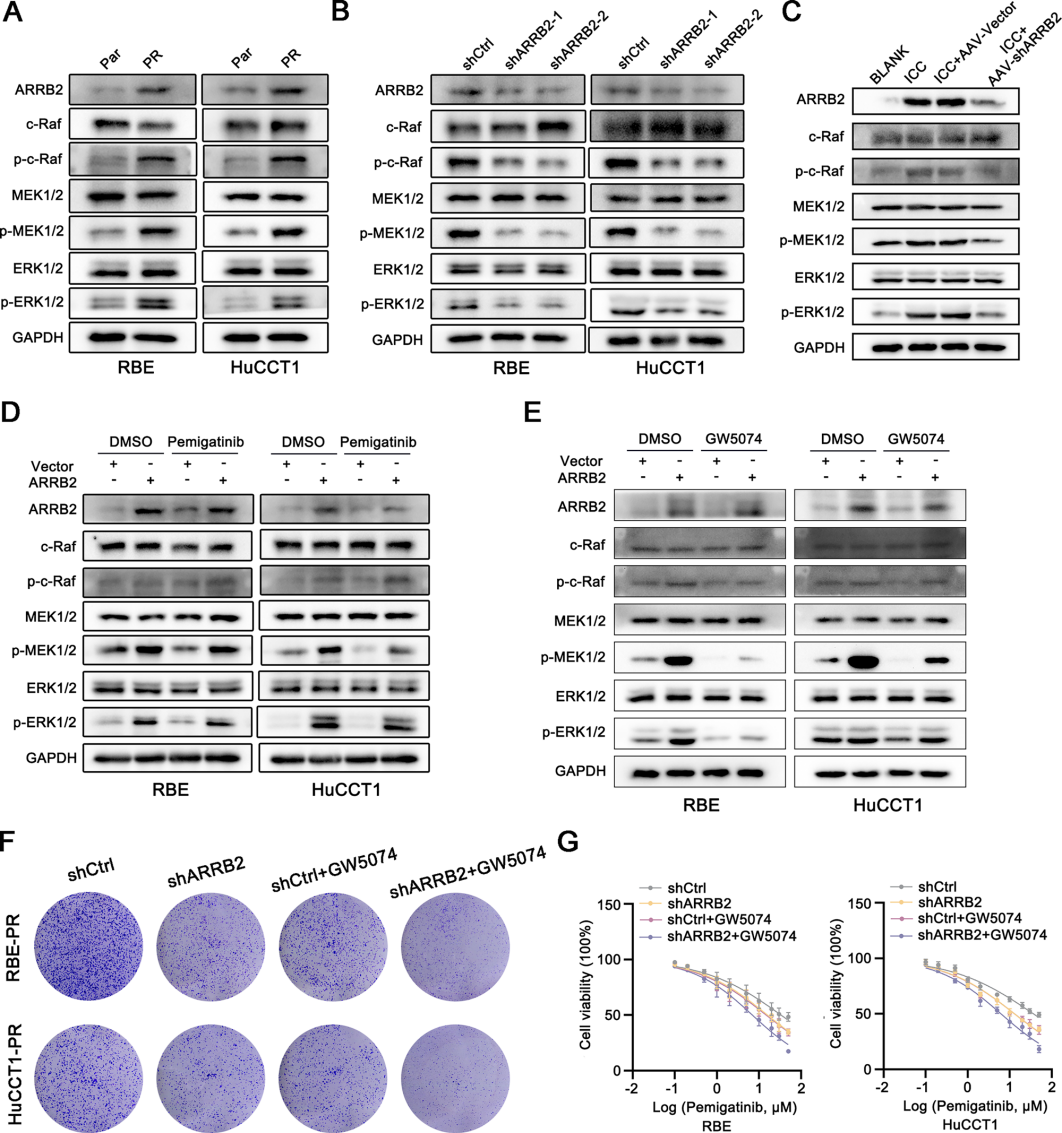
MAPK signaling pathways determine pemigatinib response in intrahepatic Cholangiocarcinoma. **A** The expressions of ARRB2, c-Raf, p-c-Raf, MEK, p-MEK, ERK and p-ERK were determined by western blot analysis between RBE-Par and RBE-PR cells (left), and HuCCT1-Par and HuCCT1-PR cells (right). **B** The expressions of ARRB2, c-Raf, p-c-Raf, MEK, p-MEK, ERK and p-ERK were determined by western blot analysis in ICC cells that were transfected with shARRB2. **C** The expressions of ARRB2, c-Raf, p-c-Raf, MEK, p-MEK, ERK and p-ERK were determined by western blot analysis in the NICD/AKT-induced ICC model following AAV8 injection. **D** RBE/HuCCT1 ARRB2-overexpressing and control cells treated with pemigatinib (10 μM) for 72 h were subjected to western blot analysis. **E** The expressions of ARRB2, c-Raf, p-c-Raf, MEK, p-MEK, ERK and p-ERK were determined by western blot analysis in ICC cells that were transfected with ARRB2-overexpressing plasmid following GW5074 treatment. **F** Colony formation in ARRB2-knockdown RBE-PR (up) and HuCCT1-PR (down) cells following GW5074 treatment. **G** Half maximal inhibitory pemigatinib concentration curves of ARRB2-knockdown RBE-PR (up) and HuCCT1-PR (down) cells following GW5074 treatment. PR pemigatinib resistant.

We further sought to identify the mediators of ARRB2-driven pemigatinib resistance. Previous studies have reported that the abnormal activation of the MAPK signaling pathway is closely associated with pemigatinib resistance [6]. Our RNA sequencing analysis revealed that the MAPK signaling pathway was enriched in ARRB2-overexpressing ICC cells (Fig. 6A, B). Notably, the expression of ARRB2 and the c-Raf/MEK/ERK axis was significantly increased in pemigatinib-resistant ICC cell lines (Fig. 7A). Furthermore, ARRB2 knockdown resulted in downregulation of c-Raf expression and subsequent inhibition of downstream signaling cascades (Fig. 7B, C). Critically, our data revealed that ARRB2 overexpression in ICC cells significantly potentiated the c-Raf/MEK/ERK signaling axis, thereby attenuating the acute inhibitory effects of pemigatinib on c-Raf/MEK/ERK phosphorylation (Fig. 7D). Furthermore, treatment with the c-Raf-specific inhibitor GW5074 effectively suppressed the c-Raf/MEK/ERK pathway (Fig. 7E) and enhanced the sensitivity of ARRB2-knockdown resistant cells to pemigatinib (Fig. 7F, G). These findings suggest a potential mechanistic role of c-Raf/MEK/ERK pathway activation in mediating resistance to pemigatinib treatment. Collectively, these data indicate that ARRB2-mediated activation of the c-Raf/MEK/ERK signaling pathway is crucial for the development of resistance to pemigatinib.

## Discussion

Despite the rapid increase in the global incidence of ICC, advancements in treatment and overall prognosis have remained stagnant over recent decades [1]. The development of individualized therapies and novel drug targets depends on advancements in genetic classification and biomarker research. Thus, further investigation into the molecular mechanisms underlying ICC pathogenesis and chemotherapy resistance is essential. Pemigatinib, a selective inhibitor of FGFR, is primarily indicated for the treatment of advanced cholangiocarcinoma with FGFR2 fusions or rearrangements [19]. Although instances of resistance to pemigatinib are frequently reported, the underlying mechanisms remain insufficiently studied [6]. Our research reveals that ARRB2 is highly expressed in pemigatinib-resistant ICC (ICC-PR) cells. Mechanistically, METTL3 promotes m6A modification of ARRB2 mRNA, while the m6A reader YTHDF1 directly binds to m6A sites on ARRB2 mRNA, thereby enhancing its stability. ARRB2 facilitates tumor progression through the activation of the MAPK and Hippo signaling pathways. Notably, the ARRB2/Raf/MEK/ERK axis is implicated in mediating resistance to pemigatinib in ICC cells (Graphical Abstract).

Increasing evidence suggests that epigenetic modifications play a crucial role in the regulation of drug resistance [20, 21]. Among these modifications, m6A is the most prevalent and evolutionarily conserved mRNA modification, significantly contributing to resistance against various treatments, including chemotherapy, radiotherapy, and immunotherapy [22–24]. Among m6A modulators, m6A writers (METTL3) and readers (YTHDF1) have been extensively studied in the progression of various cancers, including breast cancer, hepatocellular carcinoma, and colorectal cancer [25–27]. Our research showed that METTL3 augments m6A modification levels on ARRB2 mRNA. Furthermore, we found that YTHDF1 stabilizes m6A-modified ARRB2 mRNA. Additionally, ICC patients with low expression of ARRB2, METTL3, or YTHDF1 exhibited better prognosis, suggesting that these markers, in combination, may provide superior prognostic accuracy for ICC patients. Importantly, there are currently no clinically validated biomarkers available to predict the response to pemigatinib. Our findings highlight the clinical significance of ARRB2 expression in predicting outcomes of pemigatinib therapy, suggesting that ARRB2 may serve as a biomarker for assessing pemigatinib efficacy in ICC.

The MAPK signaling pathway plays a critical role in cancer progression and is frequently activated in various malignancies, including hepatocellular carcinoma [28, 29]. Similarly, the Hippo signaling pathway is essential for regulating cell proliferation and maintaining organ size, with dysregulation of the YAP-TEAD axis being associated with tumor cell proliferation, stem cell self-renewal, and tumorigenesis [30–33]. However, the role and related mechanisms of the MAPK and Hippo pathways in ICC remain inadequately characterized. In this study, our RNA sequencing analysis revealed that the MAPK and Hippo signaling pathways are involved in ARRB2-mediated ICC progression. We further elucidated that ARRB2 facilitates YAP nuclear translocation and activates the transcription of downstream target genes, thereby promoting ICC progression. Our previous research showed that the prognostically associated ICC long non-coding RNA (lncRNA-PAICC) promotes ICC cell proliferation and tumor growth through the lncRNA-PAICC-miR-141-3p/27a-3p-Yap1 axis [13]. The activation of the Hippo pathway promotes the signaling cascade associated with ICC invasion and metastasis. Additionally, our clinical investigation has identified an association between ARRB2 expression and worse prognosis in ICC patients. These findings not only provide novel mechanisms underlying the activation of the MAPK and Hippo signaling pathways but also highlight the critical role of ARRB2 in ICC progression.

Our findings that ARRB2 exerts a pro-tumorigenic role in ICC appear to contrast with a previous report suggesting a tumor-suppressive function in a mouse diethylnitrosamine-induced liver tumour model [34]. In that model, ARRB2 suppressed epithelial-mesenchymal transition (EMT) and Akt signaling, thereby attenuating metastatic potential. This contrast highlights the profound context-dependency of scaffold proteins like ARRB2. The opposing functions can be explained by examining the specific signaling pathways involved. In the cited HCC model, ARRB2 functions as a negative regulator of the pro-survival Akt pathway. Conversely, our study in ICC reveals that ARRB2 acts as a positive regulator, actively promoting the pro-proliferative Raf-MEK-ERK axis and facilitating YAP nuclear translocation to drive tumor growth and drug resistance. Notably, a pivotal study by Yang et al. demonstrated that ARRB1, but not ARRB2, is upregulated in inflammation-associated hepatocellular carcinoma and paracancerous tissues in humans, highlighting that ARRB2’s functional outcome is not intrinsic but is determined by the specific signaling network it assembles within a given cellular and pathological environment [35]. The distinct cellular origin (cholangiocytes vs. hepatocytes), etiological factors (e.g., chronic bile acid exposure in ICC), and resultant tumor microenvironment likely determine the specific GPCRs and downstream effectors (such as the Raf-MEK-ERK and Hippo/YAP pathways identified here) with which ARRB2 interacts, thereby directing its function towards either promoting or suppressing tumor growth.

Currently, therapeutic options for advanced ICC patients exhibiting resistance to pemigatinib remain limited in clinical practice [6]. Aberrant activation of the MAPK signaling pathway is significantly associated with resistance against targeted therapies [29]. In this study, we demonstrated that the activation of the ARRB2/Raf/MEK/ERK axis mediates pemigatinib resistance in ICC cells. More importantly, our results indicate that ARRB2 inhibition attenuates the activation of the Raf/MEK/ERK axis in ICC cells and restores sensitivity to pemigatinib, suggesting that the ARRB2/Raf/MEK/ERK axis plays a crucial role in the response to pemigatinib. In vitro experiments also demonstrated that ARRB2 knockdown suppressed the activation of the Raf-MEK-ERK signaling axis. Further analysis revealed that low ARRB2 expression in ICC patients is correlated with improved survival outcomes. Therefore, assessing ARRB2 expression in ICC patients to identify individuals who may benefit from ARRB2-targeted therapies warrants further clinical validation.

The advent of targeted and combination therapies has markedly enhanced treatment outcomes for ICC in recent years [36]. Elucidating the molecular mechanisms that contribute to pemigatinib resistance may improve the clinical efficacy of pemigatinib-based combination therapies for ICC patients. Our results indicate that in an in situ xenograft model, shRNA-mediated knockdown of ARRB2 expression increases the sensitivity of ICC to pemigatinib, suggesting that targeting ARRB2 may serve as a promising therapeutic strategy for overcoming pemigatinib resistance in ICC.

## Conclusions

In summary, this study highlights the biological significance of ARRB2 in the pathogenesis of ICC and the development of pemigatinib resistance. These findings suggest that ARRB2 may serve not only as a clinical biomarker for assessing pemigatinib response but also as a therapeutic target for overcoming pemigatinib resistance, thereby enhancing clinical outcomes for ICC patients undergoing pemigatinib therapy.

## Supplementary information

Supplementary Table 1

Supplementary Figure 1

Supplementary Figure 2

Supplementary Figure 3

Supplementary Figure 4

Supplementary Figure Legend

Western Blot

## Data Availability

All the data needed to evaluate the conclusions in the paper are presented in the paper and/or the Supplementary Materials. Additional data related to this paper may be requested from the authors.
