# Supplementary material for: N6-methyladenosine–mediated up-regulation of ARRB2 regulates intrahepatic cholangiocarcinoma malignant progression and pemigatinib resistance through MAPK and Hippo signaling pathways
Source: Cell Death Dis. 2026 Apr 15;17(1):508. doi: 10.1038/s41419-026-08574-8 (PMC13201787; doi:10.1038/s41419-026-08574-8)
Supplement: Supplementary file 1 — Supplementary Table 1 [file 41419_2026_8574_MOESM1_ESM.docx]

**Supplementary Table 1. Correlation between ARRB2 expression and clinicopathologic characteristics of ICC patients**

| **Characteristics** | **Cases (%)** | **Univariate analysis** | | |  | **Multivariate analysis** | | |  | **ARRB2 expression level** | | |
| --- | --- | --- | --- | --- | --- | --- | --- | --- | --- | --- | --- | --- |
|  |  | **HR** | **95% CI** | ***p*-value** |  | **HR** | **95% CI** | ***p*-value** |  | **Low** | **High** | ***p*-value** |
| **Total Cases** | 98 (100.00) |  |  |  |  |  |  |  |  |  |  |  |
| **Sex** |  | 1.46 | (0.94—2.28) | 0.095 |  | NA | NA | NA |  |  |  | **0.010** |
| Female | 35 (35.71) |  |  |  |  |  |  |  |  | 24 | 11 |  |
| Male | 63 (64.29) |  |  |  |  |  |  |  |  | 26 | 37 |  |
| **Age (years)** |  | 0.96 | (0.62—1.48) | 0.850 |  | NA | NA | NA |  |  |  | 0.232 |
| <=55 | 41 (41.84) |  |  |  |  |  |  |  |  | 18 | 23 |  |
| >55 | 57 (58.16) |  |  |  |  |  |  |  |  | 32 | 25 |  |
| **HBsAg** |  | 1.58 | (1.02—2.46) | **0.042** |  | 2.68 | (1.49—4.81) | **0.001** |  |  |  | 0.872 |
| Negative | 62 (63.27) |  |  |  |  |  |  |  |  | 31 | 29 |  |
| Positive | 36 (36.73) |  |  |  |  |  |  |  |  | 19 | 19 |  |
| **Pathological** |  | 1.61 | (1.01—2.56) | **0.045** |  | 1.94 | (1.10—3.43) | **0.023** |  |  |  | 0.155 |
| Low-grade (well/moderately) | 64 (65.31) |  |  |  |  |  |  |  |  | 36 | 28 |  |
| High-grade (poorly/undifferentiated) | 34 (34.69) |  |  |  |  |  |  |  |  | 14 | 20 |  |
| **CEA (μg/L)** |  | 1.39 | (0.87—2.23) | 0.169 |  | NA | NA | NA |  |  |  | 0.565 |
| <=5 | 70 (71.43) |  |  |  |  |  |  |  |  | 37 | 33 |  |
| >5 | 28 (28.57) |  |  |  |  |  |  |  |  | 13 | 15 |  |
| **CA-199 (U/mL)** |  | 2.26 | (1.36—3.77) | **0.002** |  | 2.89 | (1.52—5.51) | **0.001** |  |  |  | 0.145 |
| <=35 | 27 (27.55) |  |  |  |  |  |  |  |  | 17 | 10 |  |
| >35 | 71 (72.45) |  |  |  |  |  |  |  |  | 33 | 38 |  |
| **CA-125 (U/mL)** |  | 1.44 | (0.92—2.28) | 0.114 |  | NA | NA | NA |  |  |  | 0.944 |
| <=35 | 65 (66.33) |  |  |  |  |  |  |  |  | 33 | 32 |  |
| >35 | 33 (33.67) |  |  |  |  |  |  |  |  | 17 | 16 |  |
| **TBIL (μmol/L)** |  | 2.48 | (1.50—4.11) | **<0.001** |  | 1.14 | (0.39—3.32) | 0.806 |  |  |  | 0.059 |
| <=24 | 68 (69.39) |  |  |  |  |  |  |  |  | 39 | 29 |  |
| >24 | 30 (30.61) |  |  |  |  |  |  |  |  | 11 | 19 |  |
| **DBIL (μmol/L)** |  | 2.45 | (1.51—3.96) | **<0.001** |  | 1.74 | (0.71—4.26) | 0.226 |  |  |  | 0.069 |
| <=7 | 60 (61.22) |  |  |  |  |  |  |  |  | 35 | 25 |  |
| >7 | 38 (38.78) |  |  |  |  |  |  |  |  | 15 | 23 |  |
| **Tumor Number** |  | 1.25 | (0.77—2.04) | 0.366 |  | 2.40 | (1.08—5.33) | **0.032** |  |  |  | 0.291 |
| Single | 74 (75.51) |  |  |  |  |  |  |  |  | 40 | 34 |  |
| Multiple | 24 (24.49) |  |  |  |  |  |  |  |  | 10 | 14 |  |
| **Tumor Size (cm)** |  | 0.87 | (0.57—1.33) | 0.526 |  | 1.19 | (0.56—2.52) | 0.652 |  |  |  | 0.310 |
| <=5 | 48 (48.98) |  |  |  |  |  |  |  |  | 27 | 21 |  |
| >5 | 50 (51.02) |  |  |  |  |  |  |  |  | 23 | 27 |  |
| **Vascular invasion** |  | 1.67 | (1.08—2.57) | **0.021** |  | 2.08 | (0.96—4.51) | 0.062 |  |  |  | 0.160 |
| No | 52 (53.06) |  |  |  |  |  |  |  |  | 30 | 22 |  |
| Yes | 46 (46.94) |  |  |  |  |  |  |  |  | 20 | 26 |  |
| **Tumor thrombus** |  | 1.77 | (1.08—2.91) | 0.023 |  | 1.02 | (0.48—2.20) | 0.951 |  |  |  | 0.192 |
| No | 75 (76.53) |  |  |  |  |  |  |  |  | 41 | 34 |  |
| Yes | 23 (23.47) |  |  |  |  |  |  |  |  | 9 | 14 |  |
| **Satellite nodules** |  | 1.38 | (0.79—2.42) | 0.260 |  | 0.61 | (0.27—1.41) | 0.251 |  |  |  | **0.007** |
| No | 80 (81.63) |  |  |  |  |  |  |  |  | 46 | 34 |  |
| Yes | 18 (18.37) |  |  |  |  |  |  |  |  | 4 | 14 |  |
| **Lymphatic metastasis** |  | 2.82 | (1.74—4.56) | **<0.001** |  | 1.18 | (0.52—2.68) | 0.691 |  |  |  | **0.022** |
| N0 | 66 (67.35) |  |  |  |  |  |  |  |  | 39 | 27 |  |
| N1 | 32 (32.65) |  |  |  |  |  |  |  |  | 11 | 21 |  |
| **TNM-Stage (AJCC)^a^** |  | NA | NA | **<0.001** |  | NA | NA | 0.181 |  |  |  | **0.012** |
| IA | 21 (21.43) |  |  |  |  |  |  |  |  | 17 | 4 |  |
| IB | 15 (15.31) |  |  |  |  |  |  |  |  | 8 | 7 |  |
| II | 16 (16.33) |  |  |  |  |  |  |  |  | 9 | 7 |  |
| IIIA | 14 (14.28) |  |  |  |  |  |  |  |  | 5 | 9 |  |
| IIIB | 32 (32.65) |  |  |  |  |  |  |  |  | 11 | 21 |  |
| **IHC score (ARRB2)** |  | 1.23 | (1.15—1.30) | **<0.001** |  | 1.24 | (1.15—1.34) | **<0.001** |  | NA | NA | NA |

HR, hazard ratio; CI, confidence interval; TNM, tumor node metastasis; H-score, Immunohistochemistry staining score; NA, Not Applicable.

^a^ American Joint Committee on Cancer (AJCC), patients were staged in accordance with the 8th Edition of the AJCC Cancer’s TNM Classification.

The bold values indicate statistically significant differences (*P* < 0.05)
