# Supplementary figures and images for: N6-methyladenosine–mediated up-regulation of ARRB2 regulates intrahepatic cholangiocarcinoma malignant progression and pemigatinib resistance through MAPK and Hippo signaling pathways
Source: Cell Death Dis. 2026 Apr 15;17(1):508. doi: 10.1038/s41419-026-08574-8 (PMC13201787; doi:10.1038/s41419-026-08574-8)

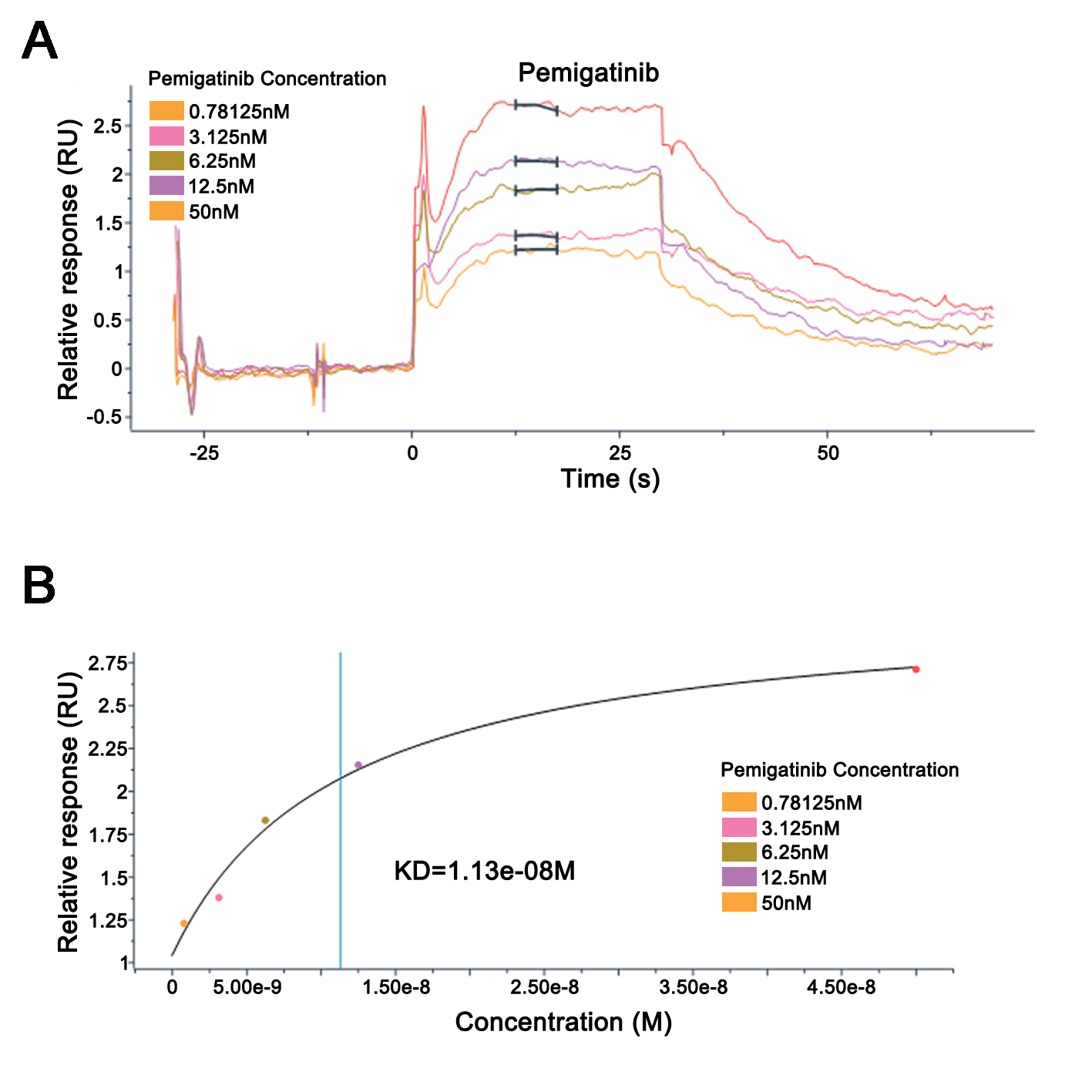

Supplement: Supplementary file 2 — Supplementary Figure 1 [file 41419_2026_8574_MOESM2_ESM.tif]

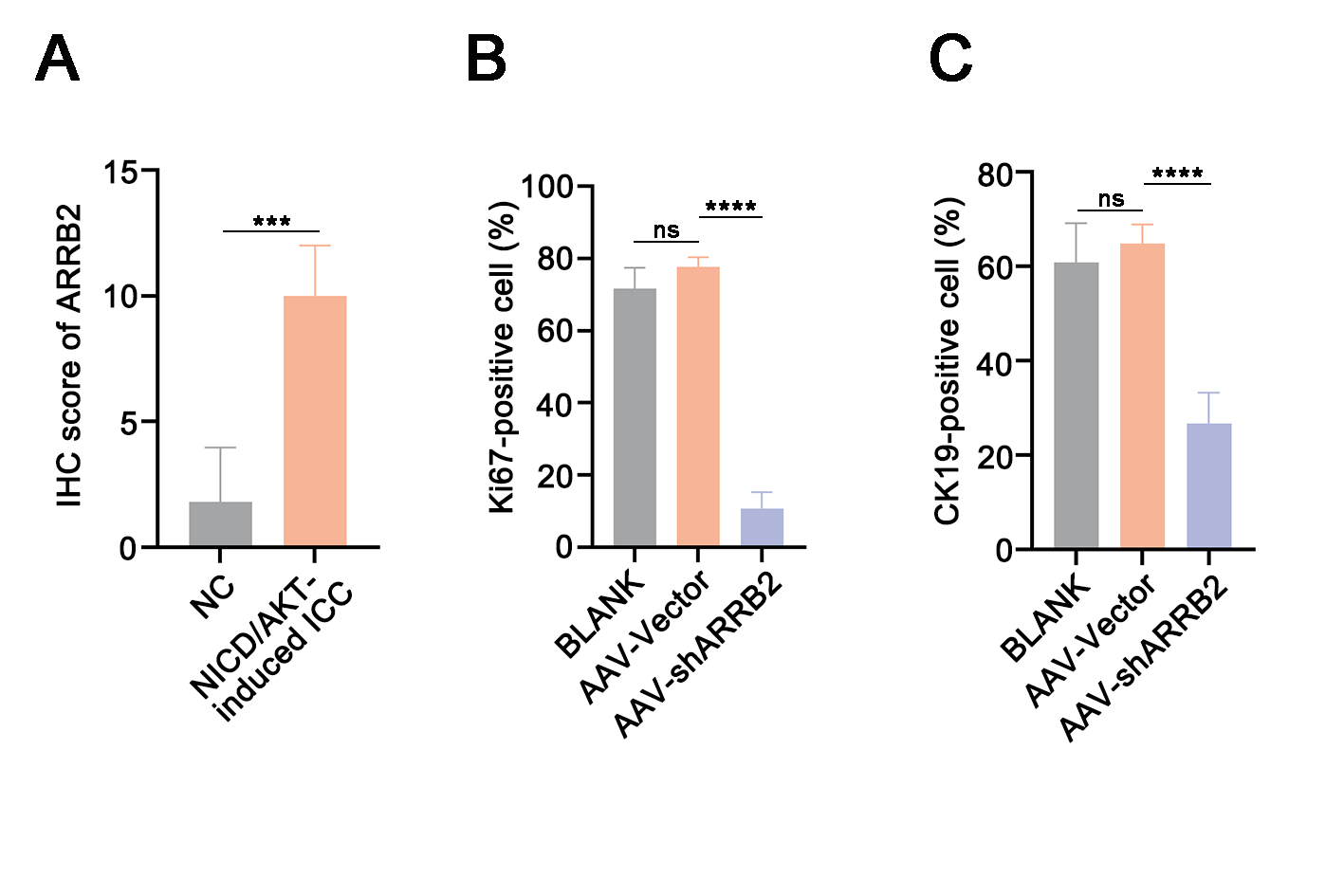

Supplement: Supplementary file 3 — Supplementary Figure 2 [file 41419_2026_8574_MOESM3_ESM.tif]

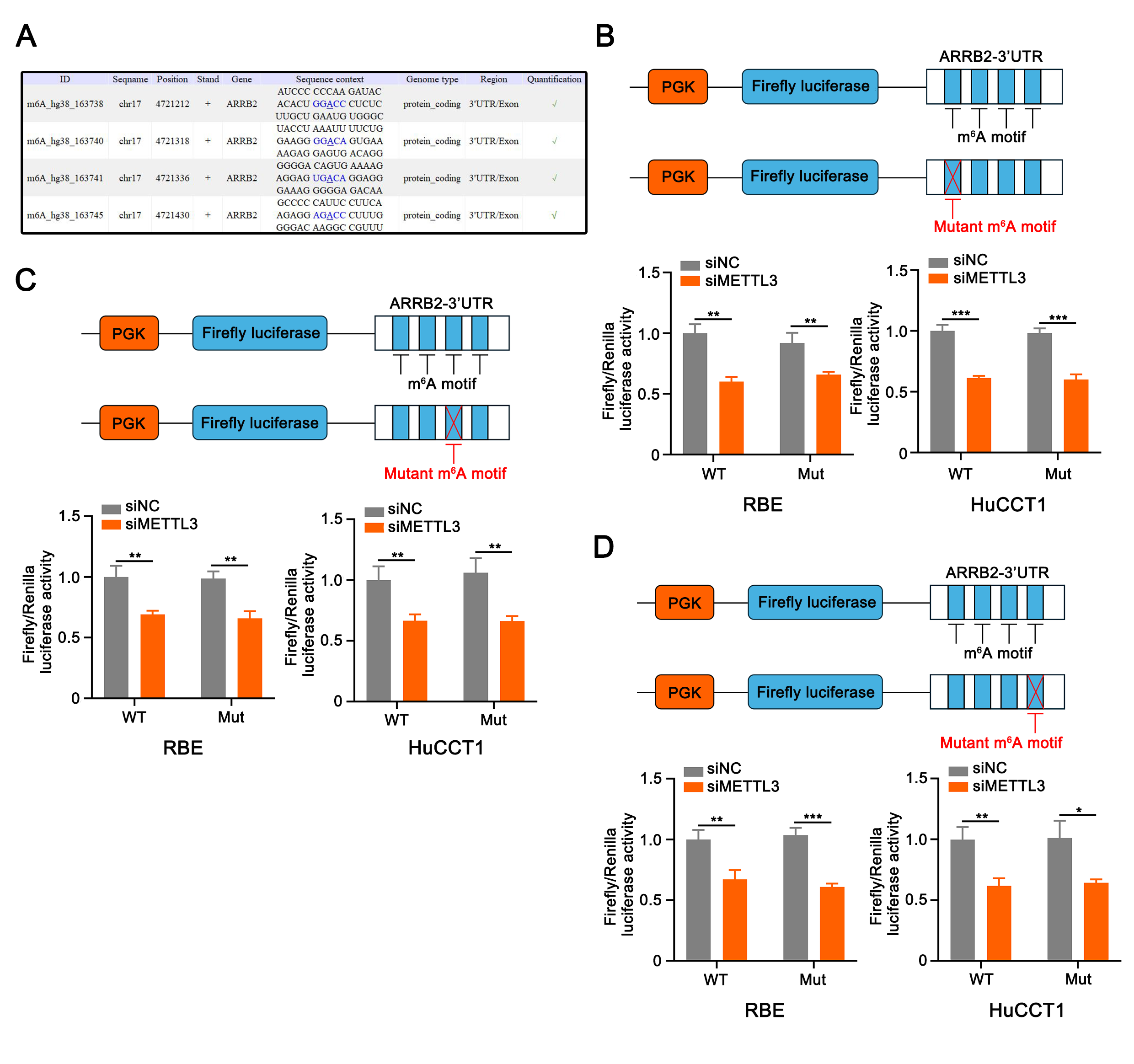

Supplement: Supplementary file 4 — Supplementary Figure 3 [file 41419_2026_8574_MOESM4_ESM.tif]

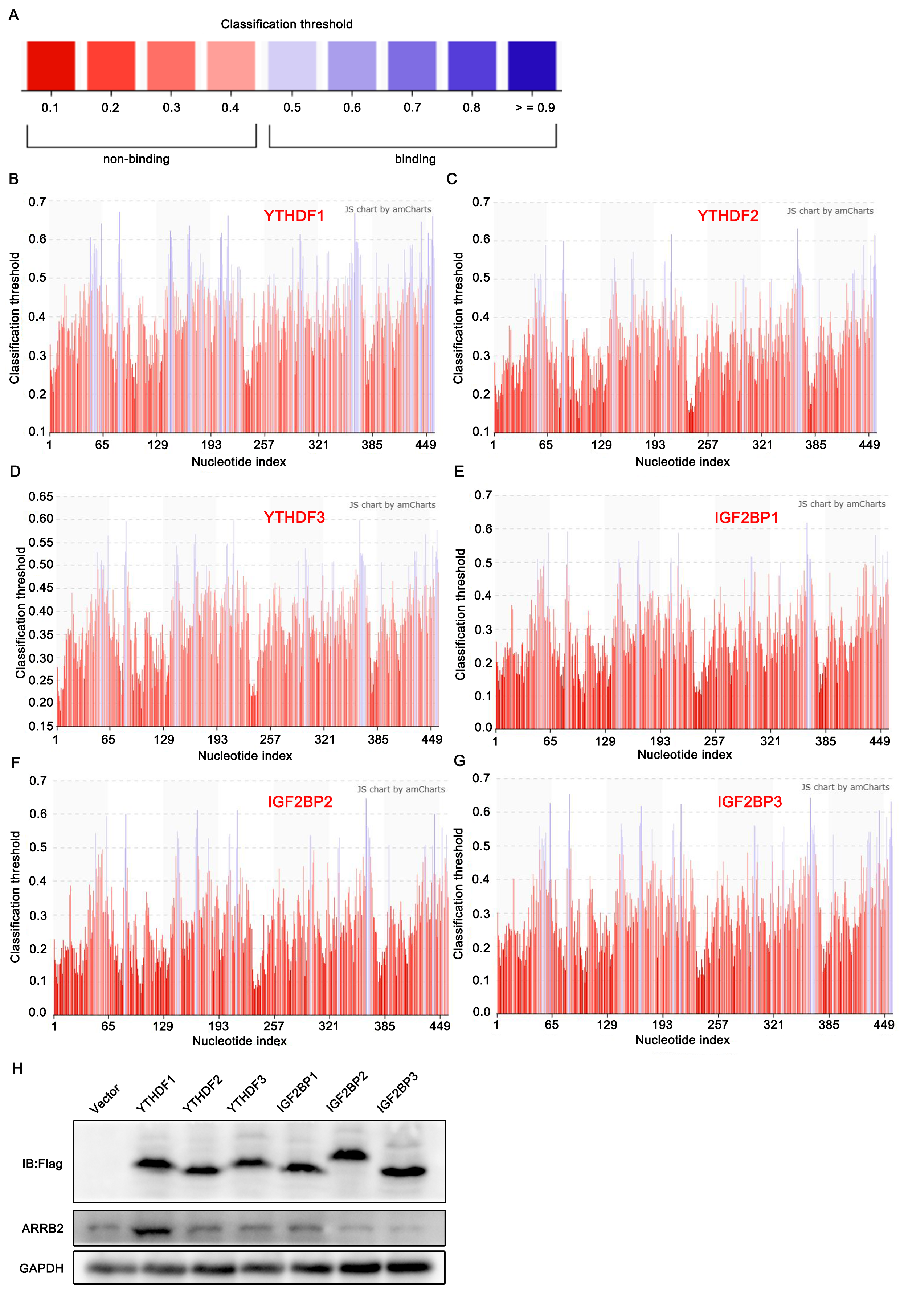

Supplement: Supplementary file 5 — Supplementary Figure 4 [file 41419_2026_8574_MOESM5_ESM.tif]

Figure 3

C

ARRB2

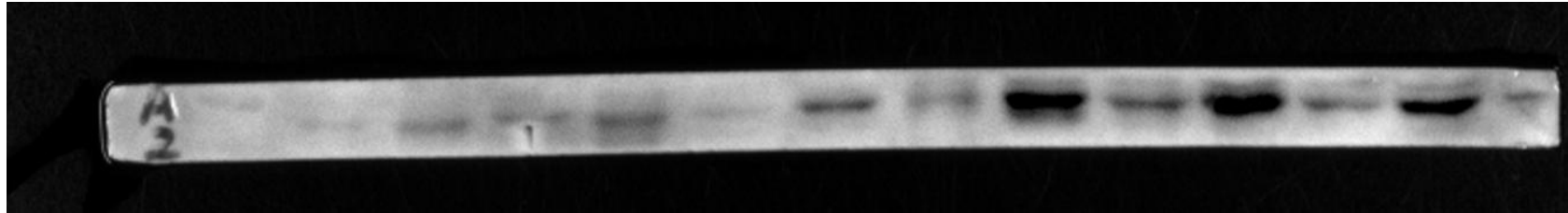

—50kDa

GAPDH

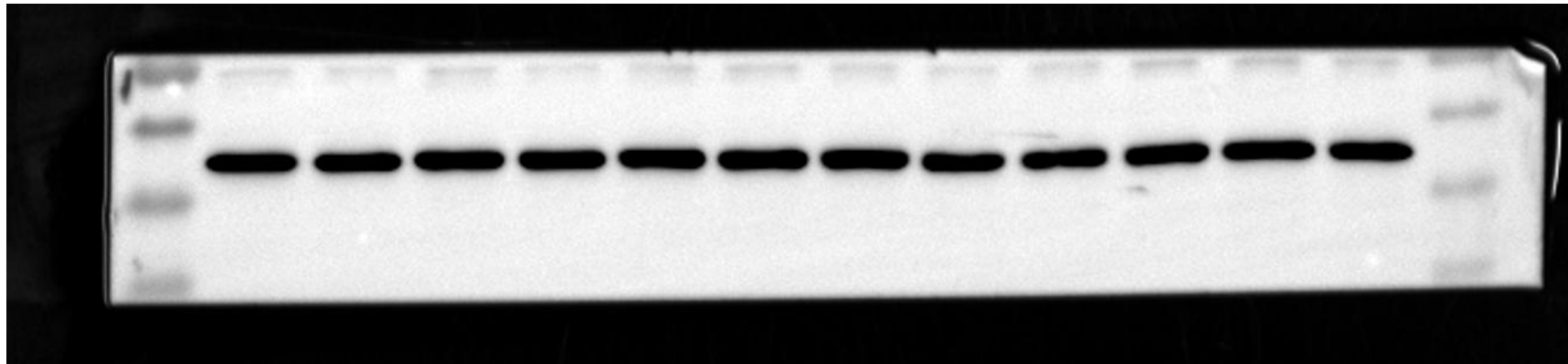

—37kDa

Figure 4

C

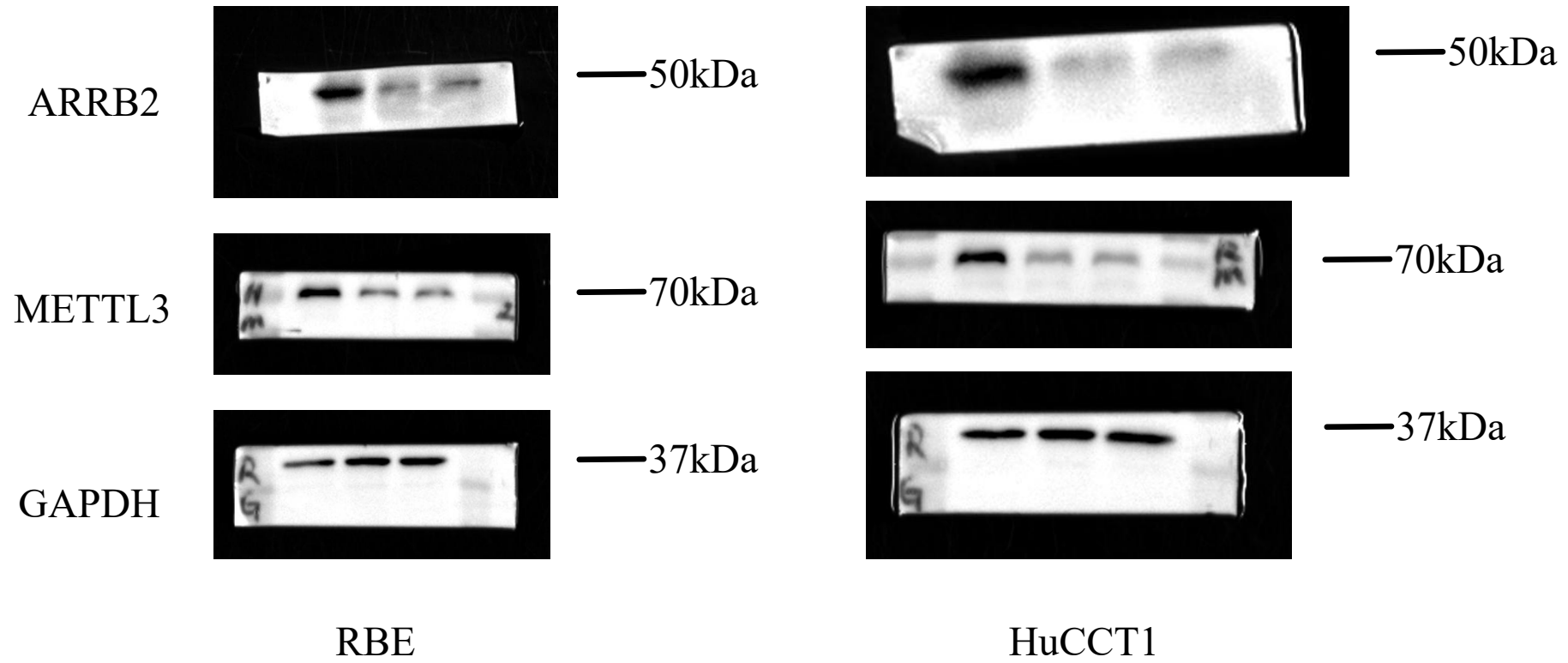

Figure 5

C

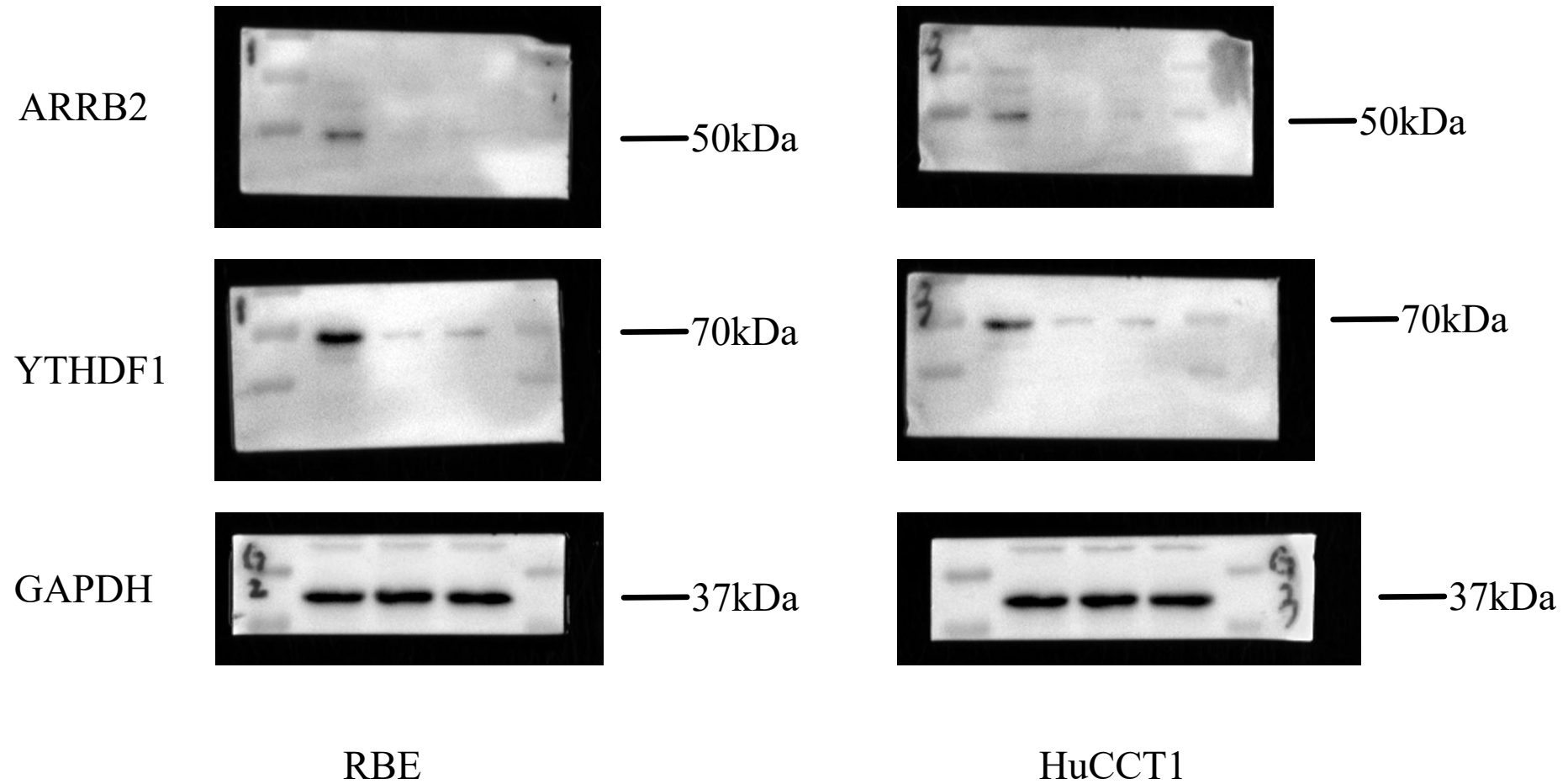

Figure 6

C

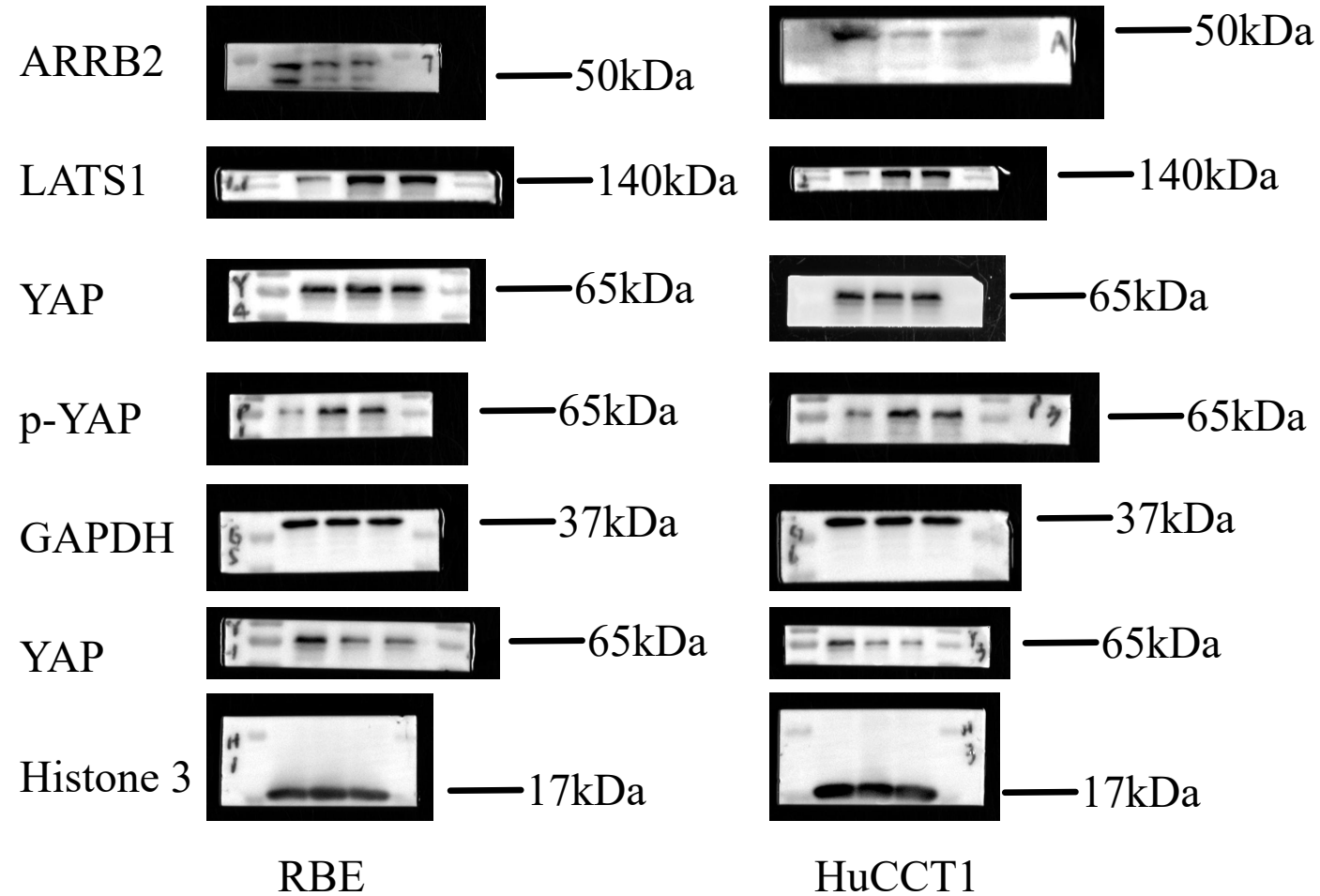

E

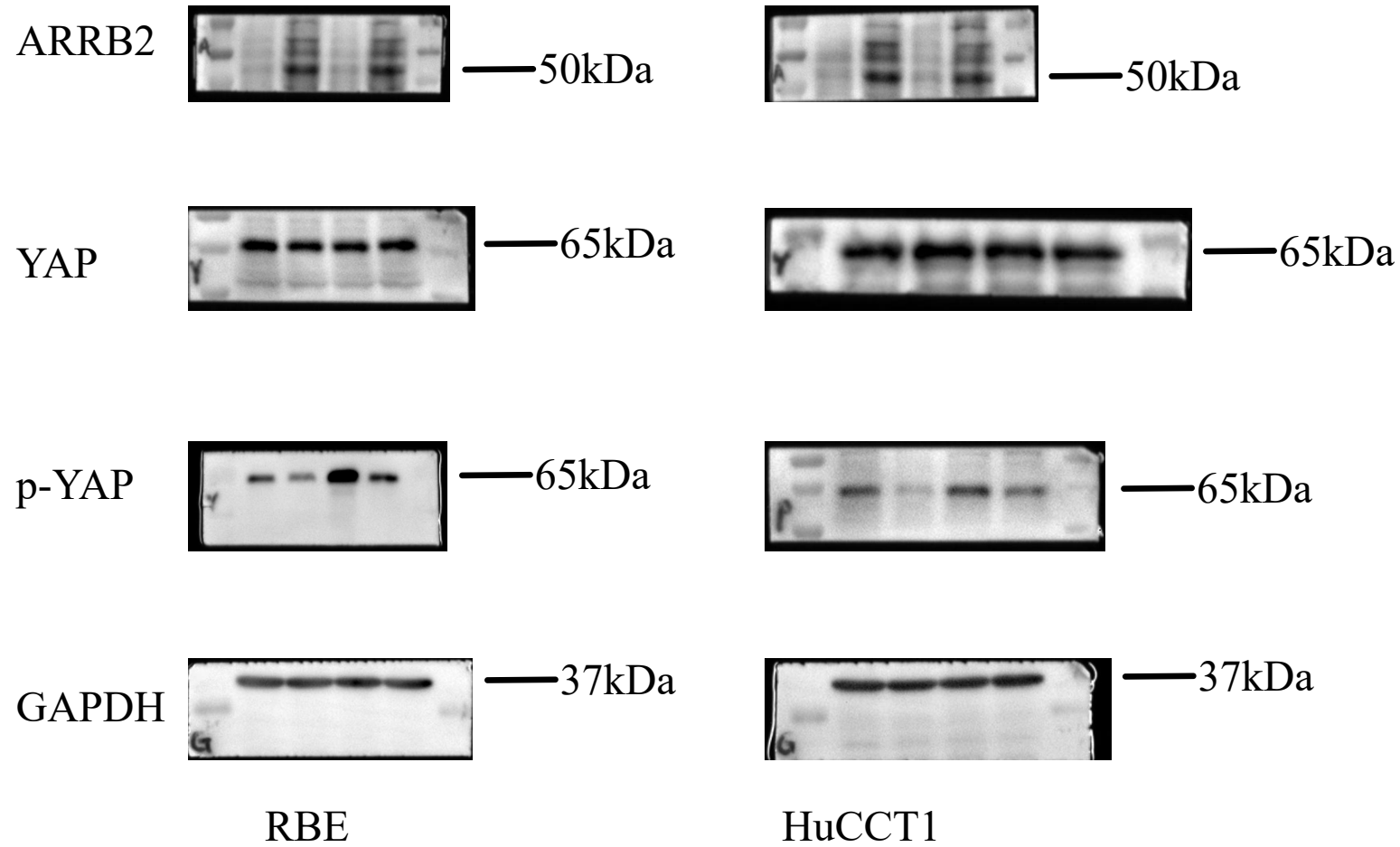

Figure 7

A

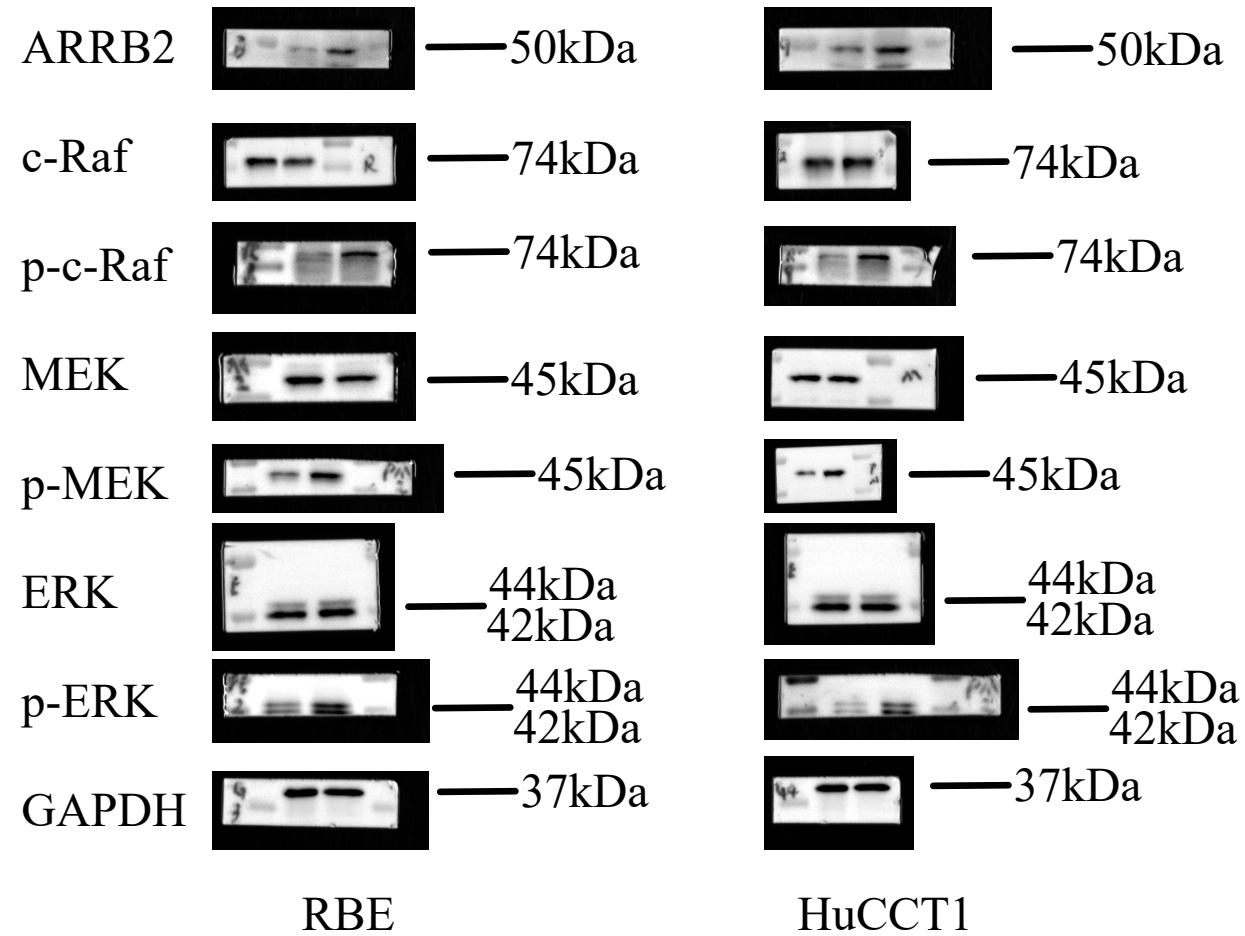

B

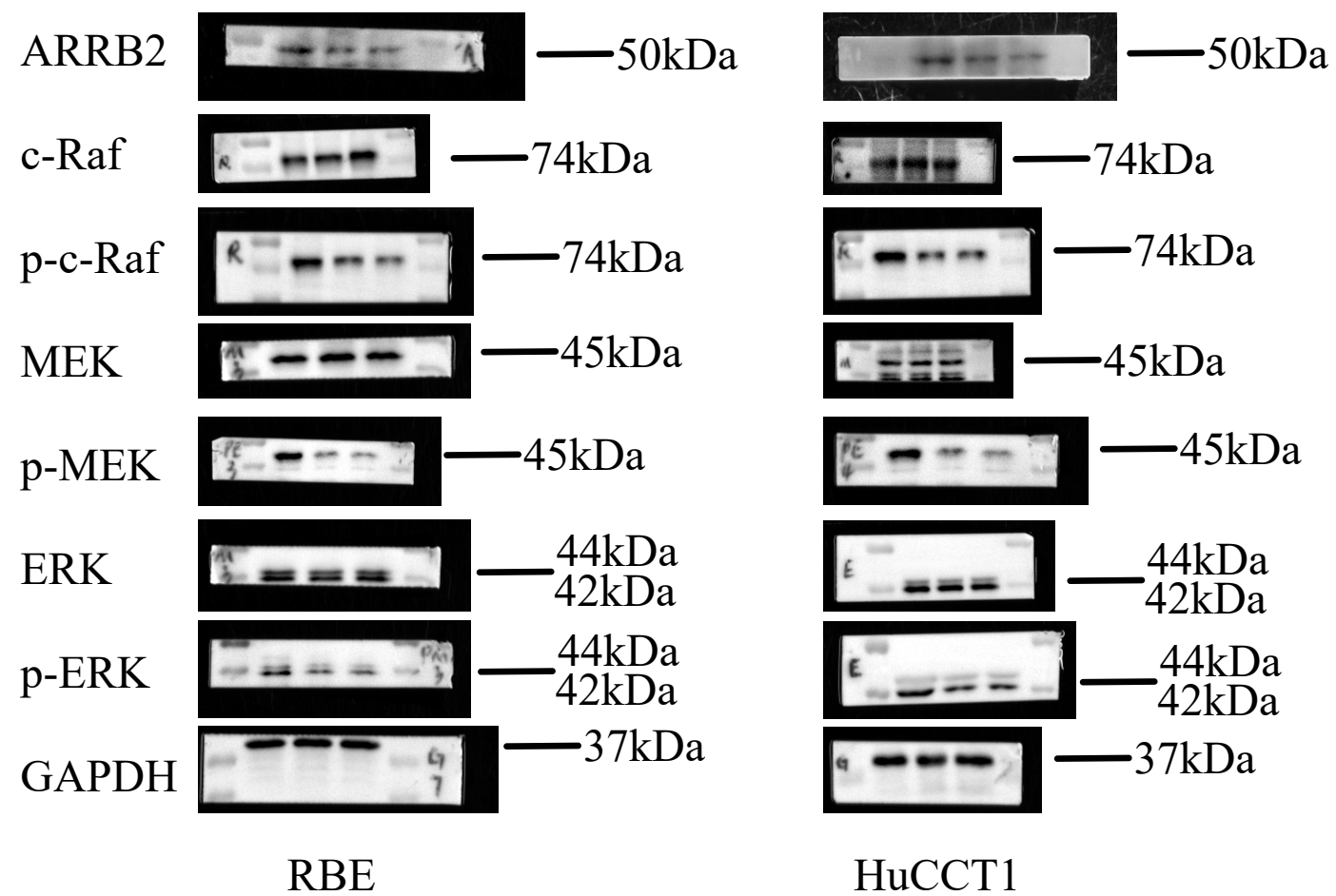

C

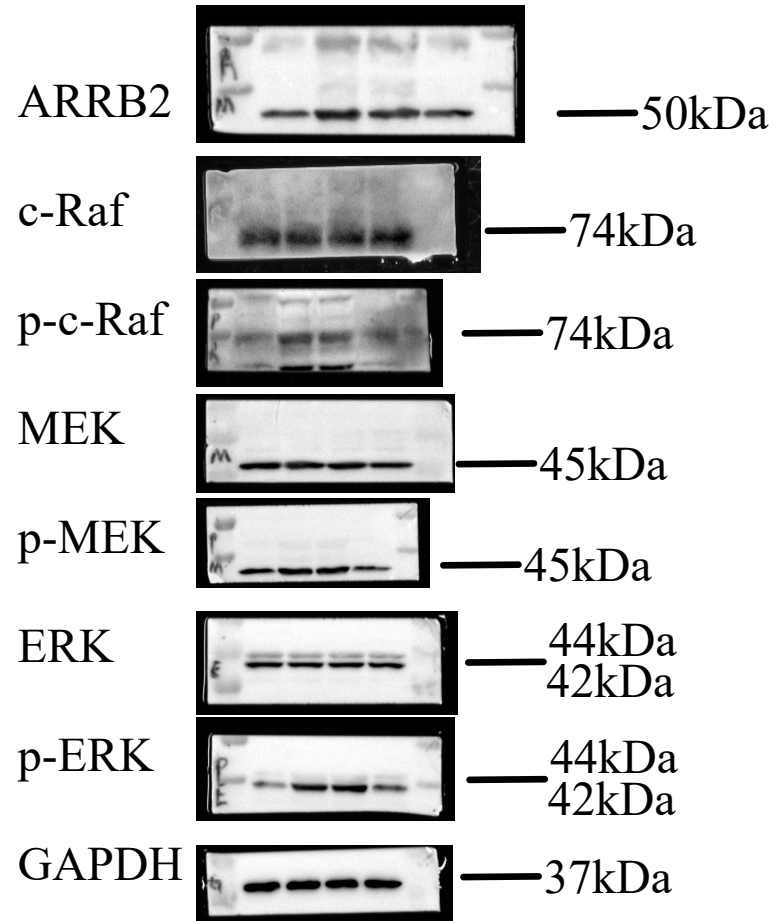

D

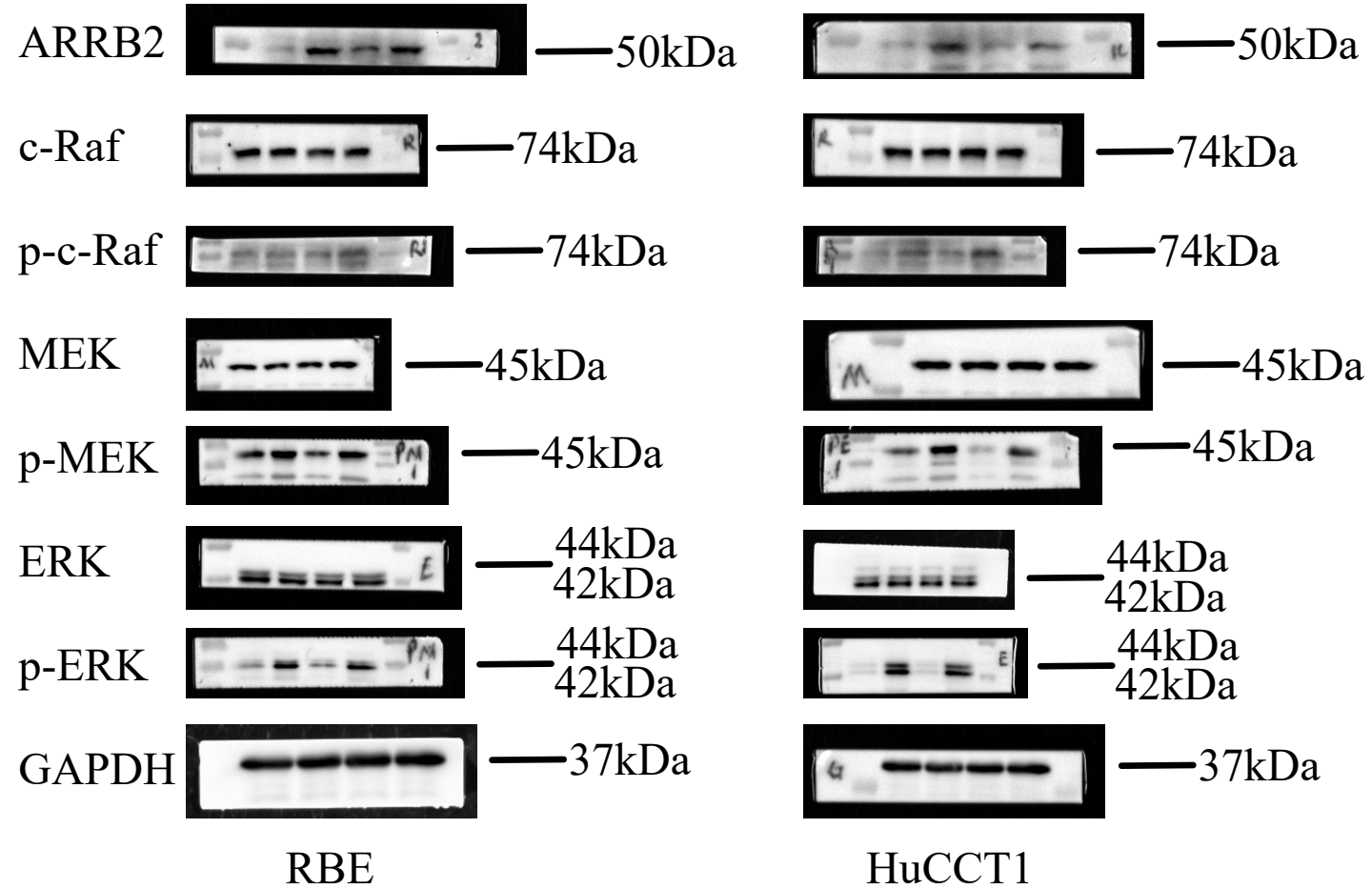

E

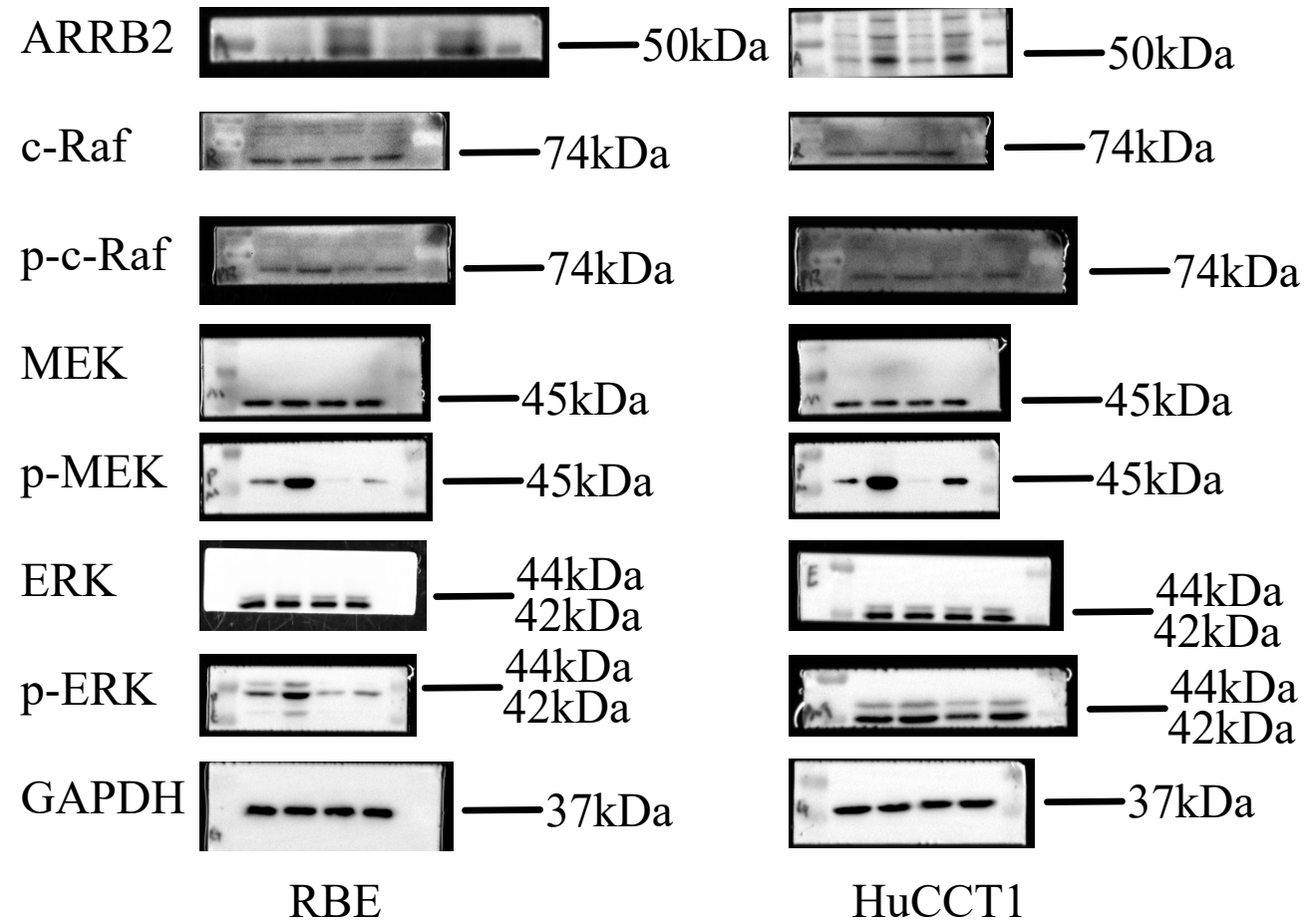

Supplement: Supplementary file 7 — Western Blot [file 41419_2026_8574_MOESM7_ESM.pdf]
