# Supplementary material for: N6-methyladenosine–mediated up-regulation of ARRB2 regulates intrahepatic cholangiocarcinoma malignant progression and pemigatinib resistance through MAPK and Hippo signaling pathways
Source: Cell Death Dis. 2026 Apr 15;17(1):508. doi: 10.1038/s41419-026-08574-8 (PMC13201787; doi:10.1038/s41419-026-08574-8)
Supplement: Supplementary file 6 — Supplementary Figure Legend [file 41419_2026_8574_MOESM6_ESM.docx]

**Supplementary Figure Legend**

**Supplementary Figure 1. ARRB2 is up-regulated in pemigatinib-resistant intrahepatic Cholangiocarcinoma cells.**

(A-B) Surface plasmon resonance sensorgrams for Pemigatinib showing the binding of FGFR2.

**Supplementary Figure 2. ARRB2 is up-regulated and predicts poor prognosis in patients with intrahepatic Cholangiocarcinoma.**

Quantification of IHC staining of ARRB2 (A), Ki67 (B) and CK19 (C) in the NICD/AKT-induced ICC model. ****P* <0.001, and *****P* <0.0001. ns, not significant.

**Supplementary Figure 3. METTL3-mediated N6-methyladenosine induces the upregulation of ARRB2 and predicts poor prognosis in patients with intrahepatic Cholangiocarcinoma.**

(A) Four m6A modification site sequencing data were collected from m6A-Atlas. (B-D) Relative luciferase activity of pmirGLO-hARRB2-3’ UTR with either wild-type or mutant (A-to-T mutation) m6A sites in ICC cells co-transfected with siMETTL3 or siNC, respectively. Firefly luciferase activity was measured and normalized to Renilla luciferase activity.

**Supplementary Figure 4. METTL3-mediated N6-methyladenosine modification of ARRB2 messenger RNA maintains its YTHDF1-dependent stability.**

(A) Classification threshold of Protein-RNA binding site prediction. (B) Bioinformatic (PRIdictor) prediction of binding sites within ARRB2 and YTHDF1. (C) Bioinformatic (PRIdictor) prediction of binding sites within ARRB2 and YTHDF2. (D) Bioinformatic (PRIdictor) prediction of binding sites within ARRB2 and YTHDF3. (E) Bioinformatic (PRIdictor) prediction of binding sites within ARRB2 and IGF2BP1. (F) Bioinformatic (PRIdictor) prediction of binding sites within ARRB2 and IGF2BP2. (G) Bioinformatic (PRIdictor) prediction of binding sites within ARRB2 and IGF2BP3. (H) WB analysis of RNA-pulldown products was conducted using Flag and ARRB2 antibodies.
